# The Transcriptomic Portrait of Locally Advanced Breast Cancer and Its Prognostic Value in a Multi-Country Cohort of Latin American Patients

**DOI:** 10.3389/fonc.2022.835626

**Published:** 2022-03-22

**Authors:** Andrea Sabina Llera, Eliana Saul Furquim Werneck Abdelhay, Nora Artagaveytia, Adrián Daneri-Navarro, Bettina Müller, Carlos Velazquez, Elsa B. Alcoba, Isabel Alonso, Daniela B. Alves da Quinta, Renata Binato, Alicia Inés Bravo, Natalia Camejo, Dirce Maria Carraro, Mónica Castro, Juan M. Castro-Cervantes, Sandra Cataldi, Alfonso Cayota, Mauricio Cerda, Alicia Colombo, Susanne Crocamo, Alicia Del Toro-Arreola, Raúl Delgadillo-Cisterna, Lucía Delgado, Marisa Dreyer-Breitenbach, Laura Fejerman, Elmer A. Fernández, Jorge Fernández, Wanda Fernández, Ramón A. Franco-Topete, Carolina Gabay, Fancy Gaete, Adriana Garibay-Escobar, Jorge Gómez, Gonzalo Greif, Thomas G. Gross, Marisol Guerrero, Marianne K. Henderson, Miguel E. Lopez-Muñoz, Alejandra Lopez-Vazquez, Silvina Maldonado, Andrés J. Morán-Mendoza, Maria Aparecida Nagai, Antonio Oceguera-Villanueva, Miguel A. Ortiz-Martínez, Jael Quintero, Antonio Quintero-Ramos, Rui M. Reis, Javier Retamales, Ernesto Rivera-Claisse, Darío Rocha, Robinson Rodríguez, Cristina Rosales, Efrain Salas-González, Verónica Sanchotena, Laura Segovia, Juan Martín Sendoya, Aida A. Silva-García, Alejandra Trinchero, Olivia Valenzuela, Vidya Vedham, Livia Zagame, United States-Latin American Cancer Research Network (US-LACRN), Juan Abarca, Osvaldo L. Podhajcer

**Affiliations:** ^1^ Molecular and Cellular Therapy Laboratory, Fundación Instituto Leloir-CONICET, Buenos Aires, Argentina; ^2^ Bone Marrow Transplantation Unit, Instituto Nacional de Câncer, Rio de Janeiro, Brazil; ^3^ Hospital de Clínicas Manuel Quintela, Universidad de la República, Montevideo, Uruguay; ^4^ Universidad de Guadalajara, Guadalajara, Mexico; ^5^ Instituto Nacional del Cáncer, Santiago, Chile; ^6^ Universidad de Sonora, Hermosillo, Mexico; ^7^ Hospital Municipal de Oncología María Curie, Buenos Aires, Argentina; ^8^ Centro Hospitalario Pereira Rossell, Montevideo, Uruguay; ^9^ Universidad Argentina de la Empresa (UADE), Instituto de Tecnología (INTEC), Buenos Aires, Argentina; ^10^ Hospital Regional de Agudos Eva Perón, San Martín, Argentina; ^11^ Laboratory of Genomics and Molecular Biology/Centro Internacional de Pesquisa (CIPE), AC Camargo Cancer Center, Sao Paulo, Brazil; ^12^ Instituto de Oncología Angel Roffo, Buenos Aires, Argentina; ^13^ Hospital de Especialidades CMNO-IMSS, Guadalajara, Mexico; ^14^ Instituto Nacional del Cáncer, Montevideo, Uruguay; ^15^ Institut Pasteur de Montevideo, Montevideo, Uruguay; ^16^ Integrative Biology Program, Instituto de Ciencias Biomédicas (ICBM), Centro de Informática Médica y Telemedicina, Facultad de Medicina, Instituto de Neurociencias Biomédicas, Universidad de Chile, Santiago, Chile; ^17^ Department of Pathology, Facultad de Medicina y Hospital Clínico, Universidad de Chile, Santiago, Chile; ^18^ Oncology Department, Instituto Nacional de Câncer, Rio de Janeiro, Brazil; ^19^ Instituto de Biologia Roberto Alcantara Gomes, Universidade do Estado do Rio de Janeiro, Rio de Janeiro, Brazil; ^20^ Department of Public Health Sciences and Comprehensive Cancer Center, University of California Davis, Davis, CA, United States; ^21^ Centro de Investigación y Desarrollo en Inmunología y Enfermedades Infecciosas [Centro de Investigación y Desarrollo en Inmunología y Enfermedades Infecciosas (CIDIE) CONICET/Universidad Católica de Córdoba], Córdoba, Argentina; ^22^ Facultad de Ciencias Exactas, Físicas y Naturales, Universidad Nacional de Córdoba, Córdoba, Argentina; ^23^ Instituto de Salud Pública, Santiago, Chile; ^24^ Hospital San Borja Arriarán, Santiago, Chile; ^25^ Organismo Público Descentralizado (OPD), Hospital Civil de Guadalajara, Universidad de Guadalajara, Guadalajara, Mexico; ^26^ Hospital Luis Tisne, Santiago, Chile; ^27^ Texas A&M University, Houston, TX, United States; ^28^ Center for Global Health, National Cancer Institute, Rockville, MD, United States; ^29^ Hospital San José, Santiago, Chile; ^30^ Hospital de Gineco-Obstetricia CMNO-IMSS, Guadalajara, Mexico; ^31^ Center for Translational Research in Oncology, Cancer Institute of São Paulo (ICESP), Sao Paulo University Medical School, Sao Paulo, Brazil; ^32^ Instituto Jalisciense de Cancerologia, Guadalajara, Mexico; ^33^ Hospital General Regional No. 1, IMSS, Obregon, Mexico; ^34^ Universidad de Sonora, Cajeme, Mexico; ^35^ Molecular Oncology Research Center, Hospital de Câncer de Barretos, Barretos, Brazil; ^36^ Grupo Oncológico Cooperativo Chileno de Investigación, Santiago, Chile; ^37^ Centro Estatal de Oncologia, Hermosillo, Mexico; ^38^ Hospital Central de las Fuerzas Armadas, Montevideo, Uruguay; ^39^ Hospital Barros Luco Trudeau, Santiago, Chile

**Keywords:** breast cancer, Latin America, PAM50 subtypes, risk of recurrence, biological pathways

## Abstract

**Purposes:**

Most molecular-based published studies on breast cancer do not adequately represent the unique and diverse genetic admixture of the Latin American population. Searching for similarities and differences in molecular pathways associated with these tumors and evaluating its impact on prognosis may help to select better therapeutic approaches.

**Patients and Methods:**

We collected clinical, pathological, and transcriptomic data of a multi-country Latin American cohort of 1,071 stage II-III breast cancer patients of the Molecular Profile of Breast Cancer Study (MPBCS) cohort. The 5-year prognostic ability of intrinsic (transcriptomic-based) PAM50 and immunohistochemical classifications, both at the cancer-specific (OSC) and disease-free survival (DFS) stages, was compared. Pathway analyses (GSEA, GSVA and MetaCore) were performed to explore differences among intrinsic subtypes.

**Results:**

PAM50 classification of the MPBCS cohort defined 42·6% of tumors as LumA, 21·3% as LumB, 13·3% as HER2E and 16·6% as Basal. Both OSC and DFS for LumA tumors were significantly better than for other subtypes, while Basal tumors had the worst prognosis. While the prognostic power of traditional subtypes calculated with hormone receptors (HR), HER2 and Ki67 determinations showed an acceptable performance, PAM50-derived risk of recurrence best discriminated low, intermediate and high-risk groups. Transcriptomic pathway analysis showed high proliferation (i.e. cell cycle control and DNA damage repair) associated with LumB, HER2E and Basal tumors, and a strong dependency on the estrogen pathway for LumA. Terms related to both innate and adaptive immune responses were seen predominantly upregulated in Basal tumors, and, to a lesser extent, in HER2E, with respect to LumA and B tumors.

**Conclusions:**

This is the first study that assesses molecular features at the transcriptomic level in a multicountry Latin American breast cancer patient cohort. Hormone-related and proliferation pathways that predominate in PAM50 and other breast cancer molecular classifications are also the main tumor-driving mechanisms in this cohort and have prognostic power. The immune-related features seen in the most aggressive subtypes may pave the way for therapeutic approaches not yet disseminated in Latin America.

**Clinical Trial Registration:**

ClinicalTrials.gov (Identifier: NCT02326857).

## Introduction

Breast cancer is a heterogeneous disease featuring distinct histological, molecular and clinical phenotypes. Although traditional classification systems leveraging clinicopathological and molecular markers are well established, most class discovery and prognostic signatures have arisen from studies including patients with European ancestry overrepresentation. For example, a recent study of the genetic ancestry of biospecimens in the The Cancer Genome Atlas (TCGA) database confirmed that only 41 breast cancer samples were classified as having Native/Indigenous American ancestry (0.4% of total samples) (1). METABRIC, another relevant breast cancer genomic resource, is composed almost exclusively of individuals of European ancestry (2).

The diversity of Latin American populations, both at the cultural and genetic ancestry levels, has an impact on the distribution of breast cancer risk. Several studies have shown differences in breast cancer incidence and mortality in the Latin American population (3, 4), in the proportion of aggressive subtypes (5), in genetic markers of risk (6) and in other epidemiological risk factors (7). Particularly for the molecular subtype distribution, most evidence is based on small, country-based cohorts or larger US-Hispanic cohorts that may not capture the diversity of Latin American populations or the impact of sociodemographic aspects [(8) and reviewed in (5)]. Thus, confirmation of the applicability and prognostic value of available intrinsic subtype classification signatures in a cohort of Latin American women is needed.

In the present study we analyzed the transcriptomic profile and comparatively assessed the prognostic performance of PAM50-based intrinsic and immunohistochemistry-based (IHC) surrogate subtype classifications in Latin American women included in the study Molecular Profiling of Stage II and III Breast Cancer in Latin American Women Receiving Standard-of-Care Treatment, in short, the Molecular Profile of Breast Cancer Study (MPBCS). Additionally, we compared the enriched molecular pathways in MPBCS subtypes with those found in the TCGA selected cohort of stage II-III breast cancer patients. This initiative of the United States-Latin America Cancer Research Network (US-LACRN, or LACRN), launched by the Center for Global Health of the US-National Cancer Institute, NIH, USA, and institutions of Argentina, Brazil, Chile, Mexico and Uruguay, focused on the integrative profiling of stage II-III breast cancer in Latin American women (9).

## Materials and Methods

### Study Setup and Patient Eligibility

After setting up consensual scientific, technical and oversight standards for research studies, LACRN launched the LACRN-MPBCS (or MPBCS) in 37 health care institutions (mostly from the public health system) and research institutes in Argentina (n=6), Brazil (n=4), Chile (n=9), México (n=13), and Uruguay (n=5), with the primary objective of characterizing the distribution of molecular profiles of stage II and III invasive breast cancer in Latin American women. The approved protocol for this study is in **Supplementary File 1**.

From 2011 to 2014, the MPBCS recruited 1449 patients (281 in Argentina, 314 in Brazil, 205 in Chile, 531 in Mexico, and 118 in Uruguay), from whom 149 were excluded due to predefined exclusion criteria or consent withdrawal, leaving 1300 eligible patients. Relevant inclusion criteria consisted of being a Latin American woman of any ethnicity residing in the recruitment countries, aged 18 years or older, with AJCC 7 clinical stage II and III breast cancer, with ECOG performance status 0-1, accessible for biopsy or candidate for primary surgery, and who have shown understanding and were willing to sign the informed consent. The exclusion criteria were prior history of non-breast malignancy up to 5 years before inclusion in the study; bilateral invasive, *in-situ*, or inflammatory breast cancer; clinical or radiological evidence of distant metastases; a prior record of hormone therapy, chemotherapy, biological therapy, targeted therapy and/or radiation therapy for breast cancer; and pregnancy and lactation. Fertile women were required to use nonhormonal contraceptives.

This constitutes a case-only, observational cohort study that was conducted in accordance with the Declaration of Helsinki and local regulations. The local Ethics Committees in each country and the NIH Institutional Review Board approved the study (IRB #: 15CN055; iRIS Reference #: 549650). All participants signed informed consent forms. This manuscript describes the primary endpoint of the study, which was the characterization of molecular profiles of breast cancer (AJCC 7 clinical stage II or III) in Latin American women with gene-expression data (n=1071). Power calculations are thus not applicable for this descriptive primary endpoint. By the study design, which included other objectives and outcomes not pertinent to this manuscript, this cohort was hospital-based and focused on tumor stages II and III (**Supplementary Information 1**).

### Clinical Procedures and Collection of Tissue Samples

Participants enrolled in the study answered a sociodemographic questionnaire and underwent a routine standard-of-care clinical, radiographic, and surgical evaluation. Clinicopathological, epidemiological and molecular data were collected in protocol-specific case report forms (CRFs) using common data elements, and according to a Manual of Operations (MOP) that was built for the study. This study used OpenClinica^®^ (openclinica.com) as the clinical data management system.

After participants signed the written informed consent form, they were enrolled in the study, their clinical evaluation was performed, and the treating physician recommended the appropriate standard of care treatment for the participant – either primary surgery or neoadjuvant chemotherapy followed by surgery – based on tumor stage, according to local institutional guidelines. Therefore, 62% of the eligible patients were treated by surgery and 38% by neoadjuvant chemotherapy followed by surgery. During neoadjuvant chemotherapy, participants received chemotherapy for a period of 16-24 weeks prior to surgery. According to protocol, the participant received treatment with four cycles of doxorubicin (60mg/m^2^) and cyclophosphamide (600mg/m^2^), every three weeks, followed by four cycles of docetaxel (90 mg/m^2^) with/without 6 mg/kg trastuzumab (8 mg/kg as loading dose) every 3 weeks or, alternatively, 12 weekly cycles of paclitaxel (80 mg/m^2^) with/without 6 mg/kg trastuzumab every 3 weeks (8 mg/kg as loading dose) for four cycles. Every three weeks the participant underwent a routine examination as directed by the physician. This included a physical examination, clinical tumor measurement, differential blood count, blood biochemistry panel, as well as a serological pregnancy test if necessary. A MUGA or echocardiography was taken at baseline, before surgery and, for participants using trastuzumab, before the start of trastuzumab associated to a taxane. Before surgery and after neoadjuvant chemotherapy, participants were re-examined by the surgeon in charge and were evaluated as to whether they were candidates for surgery (conservative or radical mastectomy). A preoperative mammogram (after the last chemotherapy administration) was done to each participant to evaluate regression or local clinical evolution. Surgery was performed within 42 days after completion of chemotherapy.

All participants had a medical follow-up every six months after surgery and a mammogram every year for five years after the date of surgery.

### Specimen Handling and Pathology Assessment

All sample collection procedures were carried out according to the LACRN consensus standard operating procedures for frozen and formalin-fixed, paraffin-embeded (FFPE) specimens based on TCGA best practice recommendations (https://brd.nci.nih.gov/brd/sop-compendium/show/701). Tissue samples were obtained from core needle biopsies or from surgical resections. Participants who were candidates for neoadjuvant chemotherapy prior to surgery underwent a routine, standard-of-care diagnostic core needle biopsy procedure for histological confirmation of breast cancer. A minimum of four and a maximum of seven 14-gauge core biopsies were collected from each participant; two to three cores were fixed in formalin (fixation time strictly in the range of 16-32 hs) for diagnostic purposes and two to four core biopsies were snap- frozen in liquid nitrogen or dry ice/acetone bath for research purposes. Cold ischemia times were strictly monitored and registered in order not to exceed 30 minutes from extraction to fixation (formalin or freezing). BSI-II (bsisystems.com) was used as the biospecimen inventory management system.

All samples were processed at the local pathology laboratory at each participating institution, using the exact same lot of commercial kits produced by the same manufacturer (Dako Products, Agilent Santa Clara, CA) and following the manufacturer’s protocol and instructions to reduce variability of results across the sites. FFPE samples were utilized for routine diagnostic work-up including histopathological evaluation of the tumor by hematoxylin and eosin (H&E) analysis and assessment of expression of tumor estrogen receptor - ER (ER pharmDx), progesterone receptor - PgR (PgR pharmDx), HER2 (HercepTest), and Ki67 (FLEX Monoclonal Mouse Anti-Human Ki-67 Antigen, Clone MIB-1) status by IHC and complemented by HER2 fluorescent *in situ* hybridization - FISH (HER2 FISH pharmDx) or chromogenic *in situ* hybridization - CISH (Her2 DuoCISH pharmDx) as appropriate (for HER2 IHC 2+ cases). ER and PgR were considered as positive when 1% or more of the tumoral cells showed positive nuclear staining (10), irrespectively of intensity, although the Allred score was also recorded (11). Ki- 67 results were recorded as % of tumoral cells with positive staining. HER2 status was evaluated and recorded as positive, negative or equivocal according to the protocol included in the MOP.

Quality control (QC) and assurance (QA) data for biospecimen handling and molecular procedures were thoroughly registered in CRFs. To ensure consistency and minimize variations, intracountry evaluations were performed for IHC data obtained with ER, PgR, Ki67 and HER2 and *in situ* hybridization using either FISH or CISH, as appropriate. In addition, reference centers in all countries went through an external evaluation by the College of American Pathologists for IHC (ER, PgR), and FISH and CISH HER2 testing. A specific analysis of the performance of the Ki67 determination was done to evaluate variation between centers (**Supplementary File 2** - Extended Methods).

### MPBCS Expression Data Acquisition and Pre-Processing

Two-color microarray analysis was performed using the Agilent gene expression platform (Series C Scanner, Agilent, Santa Clara, CA), processed in central molecular biology laboratories located in Argentina (Instituto Leloir), Brazil (Instituto de Câncer do Estado de Sao Paulo, Hospital do Câncer de Barretos, AC Camargo Cancer Center and Instituto Nacional de Câncer de Brasil), Mexico (University of Guadalajara and University of Sonora), Chile (Instituto de Salud Pública de Chile) and Uruguay (Instituto Pasteur). All laboratories were trained during a dedicated workshop and followed the same SOPs for total RNA isolation, quantification, labeling, hybridization, and scanning to ensure proper harmonization and minimize bias. An external quality control of results was performed by Dr Katherine Hoadley at University of North Carolina.

Snap-frozen tissue samples collected during biopsy or surgery before any chemotherapy were utilized to extract RNA. If both biopsy and surgical resection samples were available from a participant, RNA was extracted from the surgical sample. Only tissues with tumor content higher than 60%, as evaluated by histopathology, were processed.

Total tumor RNA was extracted from each tissue sample using RNeasy Mini Kit (QIAGEN, Hilden, Germany). RNAs were quantified using NanoDrop 2000 and RNA integrity was evaluated on a 2100 Bioanalyzer (Agilent Technologies, Santa Clara CA, USA). A RIN higher than 6.0 was considered as of enough quality to perform the microarrays.

Tumor RNA and a Universal Human Reference RNA (Stratagene, San Diego CA, USA) were amplified, differentially labeled with Cy5 and Cy3, respectively, using Agilent Low Input Quick Amp Labeling Kit 2-Color, and subsequently hybridized 1:1 in mass (i.e. 825 ng each labeled RNA) with Human Gene Expression v2 4x44K (AMADID 026652) microarrays using the Agilent Gene Expression Hybridization Kit and Wash Buffer (Agilent Technologies, Santa Clara CA, USA). Feature Extraction 11.5.1.1 software (Agilent Technologies, Santa Clara CA, USA) was used by all laboratories to generate raw data. Raw data were subsequently analyzed in a single batch. Microarrays with signal intensities below 10,000 for 99% of signal distribution were considered failed. Samples with ‘out of range’ Feature Extraction QC evaluation metrics or with lower signal intensity (<25,000) were flagged for later analysis. Expression data for probes was processed as log2 ratios, probes were mapped to Entrez Gene IDs according to HsAgilentDesign026652.db R package and probes without a valid Entrez Gene ID were removed from further analysis.

A total of 229 patients (17%) failed to render reliable gene expression data due to one of the following quality issues: failure at obtaining biopsy, insufficient tumor content, poor RNA quality and/or quantity, poor array hybridization or poor quality metrics after array scanning.

### TCGA Data Acquisition and Processing

TCGA clinical information and PAM50 classification limited to primary tumor samples stages II-III were obtained from cBioPortal (12) on 2020-04-22. RNA-seq raw counts from the stages II-III subset of breast cancer tumors of the TCGA RNAseq dataset (TCGArf) were downloaded with TCGA-Assembler pipeline (13) on 2018-01-12. Normalization factors were calculated using the trimmed mean of M-values (TMM (14),). For plotting and GSEA analysis, raw count data was transformed to log2 counts per million with a prior count of 0.5. For differential expression analysis, raw counts were processed by *voom* (15) previous to *limma* (16) analysis. Gene symbols were obtained from the available Entrez IDs using *org.Hs.eg.db* R library (Carlson M (2019). org.Hs.eg.db: Genome wide annotation for Human. R package version 3.8.2.), and genes for which no valid symbol could be found were removed from the databases.

### PAM50 Subtyping and Risk of Recurrence Score (ROR) Calculation

The expression log2 ratios of the PAM50 genes were centered according to their balanced medians. To obtain the balanced medians, the dataset was subsampled to match the ER distribution of that in the training set used for PAM50. Balanced ER-subsets were identified in each country; for each country we identified the maximum number of high-quality ER-negative samples (the limiting factor), and then randomly selected a similar number of ER-positive samples. A total of 248 ER-negative and 248 ER-positive samples were selected to determine an ER-balanced median for each of the 50 genes used in the PAM50 subtyping scheme. This included pairs from each country (Argentina 59, Brazil 46, Chile 32, Mexico Guadalajara 73, Mexico Sonora 22, and Uruguay 16 pairs). The balanced medians were then used to center the gene expression data of the whole cohort and the PAM50 algorithm was ran as suggested (17) (generic code - https://genome.unc.edu/pubsup/breastGEO/PAM50.zip). Risk of Recurrence score – subtype only (ROR-S) was calculated as described in (17).

### Pathway Enrichment and Differential Expression Analysis

For pathway analysis, additional QC and normalization of gene expression data were performed using the R package Agi4x44.2c (18). For the MPBCS cohort a batch correction was done due to a technical bias introduced by the year in which the microarray analysis was performed (**Supplementary File 2** – Extended Methods). For this, prior to averaging probe expression by gene symbol, two batches based on processing dates (i.e. before or after September 2017) were defined for the whole cohort. Probe expression data was normalized to equalize median-absolute values and adjusted for batch effects using the ComBat function of sva package (v 3.32.1) with default settings and the earlier batch as reference.

Pathway enrichment was studied by GSEA and GSVA using a single list of 1200 curated gene sets comprising MSigDB Hallmark, KEGG and REACTOME, that contain between 15 and 500 genes, identified by Entrez ID (19, 20). GSEA was performed using GSEA desktop software v 4.0.3 with standard settings, considering a *P*-value <0.05 as significant. For microarray data, GSVA was applied on the expression data normalized to equalize median-absolute values, using the Gaussian method to estimate the cumulative distribution function. For RNA-seq data, GSVA was applied on raw counts data, using the Poisson method to estimate the cumulative distribution function. All comparisons with their GSEA normalized enriched score (NES) or GSVA coefficient, *P-* and adjusted *P*-values are detailed in **Supplementary Table 1**.

Collapse of enriched terms (gene sets) was performed for each contrast by 1) selecting the top ten significant (i.e. p<0.05) gene sets with highest GSEA NES absolute values, 2) verifying that those terms were also significant in GSVA (i.e. adjusted *P*-value <0.05, 3) reviewing the rest of significant terms in GSEA analysis using as keywords genes already deemed relevant in the literature for the corresponding intrinsic subtype contrast, and 4) manually identifying common terms and pathways in the resulting list of terms (**Supplementary Table 1**, see References worksheet).

Pathway analysis with differentially expressed genes were performed using MetaCore™ software (Clarivate Analytics, USA) (https://portal.genego.com/). First, normalized gene expression values of TCGA and MPBCS cohorts were used to obtain a list of differentially expressed genes (DEGs) for each contrast (e.g. luminal A vs basal-like and so on) using *limma* (16). A batch identification variable was included in the model as a covariate. DEGs for each contrast were defined as those with log fold-change (logFC) >0.5 and a Benjamini-Hochberg adjusted *P*-value<0.05. With both MPBCS and TCGA lists of DEGs, we identified those DEGs common to both cohorts and those exclusive to each cohort using http://bioinformatics.psb.ugent.be/webtools/Venn/. Lists of common, MPBCS-exclusive and TCGA-exclusive DEGs were then used for independent MetaCore analyses. Hierarchical clustering with Spearman distance with selected genes was performed using *ComplexHeatmap* R package (21).

### Transcription Factor Activity Analysis

Transcription factors (TF) activities per sample were inferred from gene expression data using DoRothEA in combination with the statistical method VIPER as it incorporates the mode of regulation of each TF-target interaction (22). DoRothEA is a gene set resource containing signed TF-target interactions curated and collected from different types of evidence. Each interaction is accompanied by an interaction confidence level ranging from A to E. We used the level A as it is the highest reliable level according to the authors.

The multi-unpaired comparisons between PAM50 subtypes were computed using the *limma R* package. The TF activities per sample obtained from DoRothEA-VIPER were used to fit a linear model for each TF and the *eBayes* test used to obtain the corresponding adjusted *P* values and the logFC as described in (23).

### Determination of the Cytolytic Score (CYT)

CYT was calculated from the microarray batch-normalized expression matrix as the geometric mean of granzyme A (*GZMA*) and perforin (*PRF1*) expression (24).

### Classification of TNBC Tumors in Lehmann’s Molecular Subtypes (TNBC-6) and the Derived TNBC-4 and Bareche-2018

TNBC tumors were defined as those HR-/HER- (n=170). Lehmann’s molecular subtypes (TNBC-6) were assigned to the TNBC samples using the TNBCtype online subtyping tool (http://cbc.mc.vanderbilt.edu/tnbc/) (25). Samples in which ER expression was greater than the 75 percentile at transcriptome level were removed, leaving a total of 145 samples for analysis. Each TNBC sample was assigned to a TNBC molecular subtype based on the highest Pearson correlation (centroid) and lowest p-value. Those samples with low correlation with all centroids (i.e. correlation coefficient <0.1 or P-value <0.05) or whose correlation coeficients were similar between subtypes (difference of two largest correlation coefficients <0.05) would be considered unclassified (UNS). A total of 128 tumors were assigned to any of the TNBC-6 classes.

For the TNBC-4 reclassification, IM and MSL samples were re-assigned to the second highest correlated centroid (26). For Bareche-2018 classification, BL2 samples were re-assigned to the second highest correlated centroid (27).

### Statistical Analysis

Association among different clinicopathological variables was tested for significance with the Chi-square test, and Cramer’s V test provided a measure of the strength of the association (28). Survival analyses were performed using the Kaplan–Meier estimator (*survival* R package), comparing each pair of curves with the log-rank test. Overall cancer-related survival (OSC) was defined as the interval from informed consent signature to the date of death. Disease-free survival (DFS) comprises the interval from date of surgery to first recurrence with medical confirmation, respectively. Disease-free patients were censored at the time of the last follow-up. For patients known to have a progression but missing an exact progression date, the date of the last medical examination was used. For each risk classification (i.e. PAM50, IHC, IHC-St. Gallen, etc) and each survival response (OSC and DFS), univariate and multivariate Cox proportional-hazards models were fitted with all available data points, and p-value and Harrels’ correlation index were calculated.

Comparison of medians was performed using the Kruskal-Wallis test with Dunn’s post test for sample pair comparison. P values were corrected for multiple comparisons using the Benjamini-Hochberg adjustment.

## Results

### Clinical Features of MPBCS Samples According to the PAM50 Classifier

**Figure 1:**
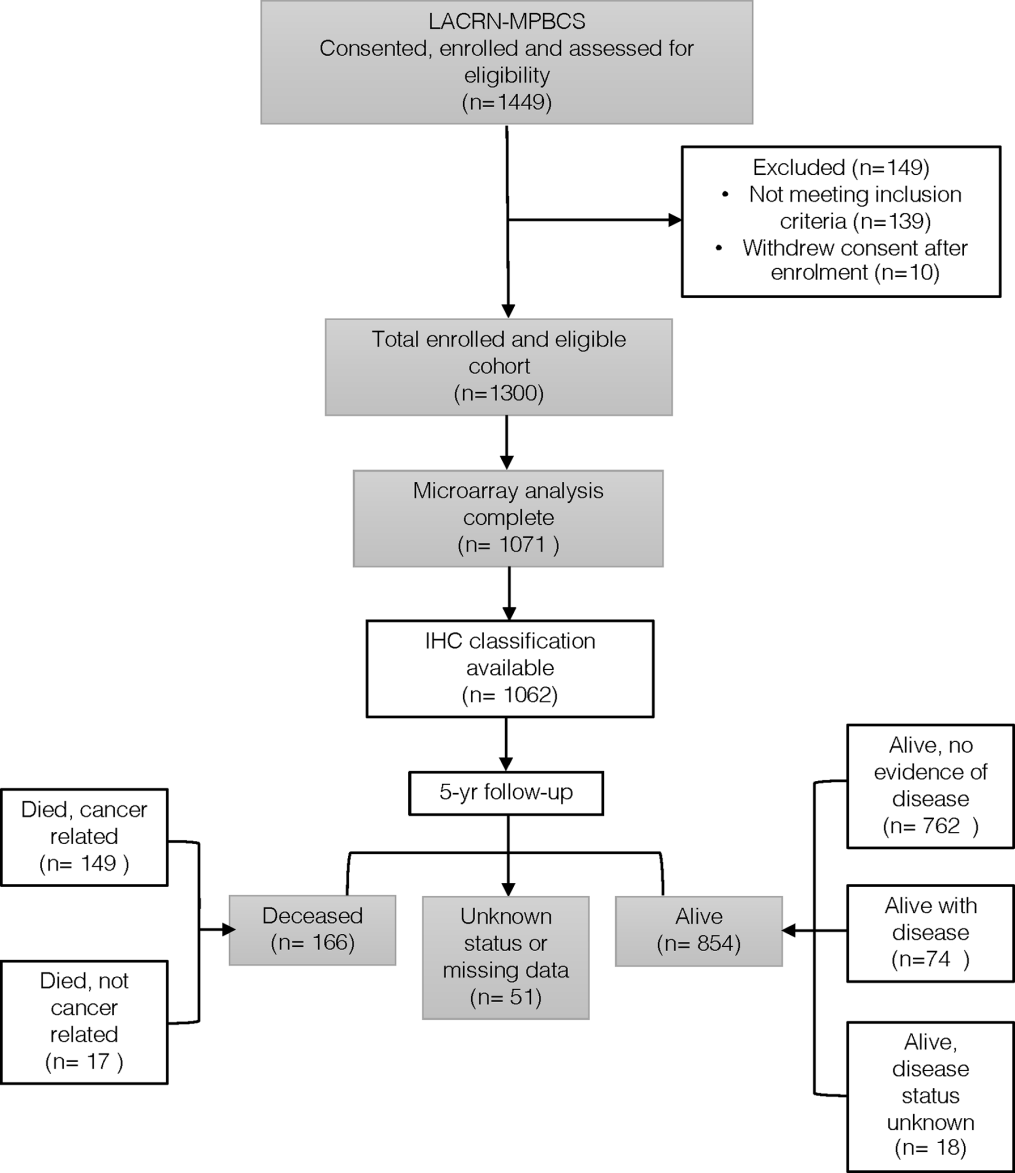
Flow diagram of the MPBC Study.

From the 1300 eligible MPBCS patients, a total of 1071 tumors were successfully characterized by gene expression microarrays and constituted the core of this work (**Figure 1**). A unique characteristic of this study was that each site performed gene expression analysis independently with their own equipment and personnel. The harmonization of all procedures related to sample obtention, storage, RNA extraction and microarray analysis was critical to minimize batch effects in microarray analysis. Using the PAM50 genes, principal component analysis (PCA) showed no significant bias by country, the arm of the study, type of sample (i.e., biopsy or surgical specimen) or year of microarray hybridization, confirming the data reliability (**Supplementary Figure S1**) and allowing to classify patients according to gene expression-derived PAM50 intrinsic subtypes without any batch correction.

PAM50 classification defined 456 tumors (42.6%) as luminal A (LumA), 228 (21.3%) as luminal B (LumB), 142 (13.3%) as HER2-enriched (HER2E) and 178 (16.6%) as basal-like (Basal). Sixty-seven tumors (6.3%) were classified as normal-like and were therefore excluded from some of the analyses. Normal-like tumors are considered tumors with very little tumor content and with no prognostic value (17, 29). Moreover, as there is no surrogate category for Normal-like tumors, it was not possible to perform any comparison with other prognostic classifications.

**Table 1** shows a summary of the clinical characteristics of the 1071 tumor samples and their distribution according to PAM50 subtypes. Age was significantly associated with the PAM50 subtype (p<0.001), as a significantly lower mean age at diagnosis was seen for Basal (50.4 +/- 10.8 years) cases than for the rest of the subtypes (except normal-like). All clinical and pathological data were associated to a certain degree with PAM50 subtyping. As shown by the color shades in **Table 1**, histological type, clinical stage, tumor size and lymph node status, albeit significantly associated, showed relatively low correlations with PAM50 subtypes. On the other hand, tumors larger than 50 mm in size and/or with lymph node metastasis were enriched in non-LumA subtypes. IHC-based ER, PgR and FISH-based HER-2 status were strongly correlated with PAM50 subtypes while Ki67 values above the 20% threshold were moderately correlated with non-LumA subtypes (**Table 1**).

**Table 1:** Summary of clinicopathological characteristics in the MPBCS cohort and their association with PAM50 subtypes^#^.

Clinical parameter	Whole cohort n (%)	PAM50					Cramer’s V/AOV
		LumA	LumB	Her2	Basal	Normal	
**Number of subjects (%)**	1071	456 (42.6%)	228 (21.3%)	142 (13.3%)	178 (16.6%)	67 (6.3%)	
**Age at diagnosis - mean (SD)**	54.5 (12.2)	56.4 (12.4)a	55.4 (13.4)a	54.3 (11)a	50.4 (10.8)b	50.3 (9.6)b	p < 0.001
**Histological type**							0.1 (low)
Invasive (infiltrating) lobular carcinoma	62 (5.8%)	40	10	2	2	8	
Invasive (inflitrating) ductal carcinoma	919 (85.8%)	372	197	134	162	54	
Invasive mammary carcinoma (NOS)	19 (1.8%)	10	6	0	3	0	
Invasive mixed ductal and lobular carcinoma	24 (2.2%)	13	7	0	1	3	
Other	47 (4.4%)	21	8	6	10	2	
**Histological grade**							0.26 (medium)
Low	128 (12%)	100	10	5	4	9	
Intermediate	473 (44.2%)	240	113	48	43	29	
High	431 (40.2%)	98	94	85	130	24	
Missing	39 (3.6%)	18	11	4	1	5	
**Clinical stage**							0.14 (low)
II A	383 (35.8%)	209	80	42	34	18	
II B	324 (30.3%)	137	69	41	57	20	
III A	199 (18.6%)	39	48	38	59	15	
III B	98 (9.2%)	40	21	14	15	8	
Missing/Other	67 (6.3%)	31	10	7	13	6	
**Lymph node status**							0.15 (low)
Positive	556 (51.9%)	191	120	84	123	38	
Negative	482 (45%)	250	102	52	50	28	
Missing	33 (3.1%)	15	6	6	5	1	
**Tumor size at diagnosis**							0.12 (low)
< = 20mm	113 (10.6%)	60	22	10	13	8	
> 20-50mm	666 (62.2%)	311	140	83	98	34	
> 50mm	257 (24%)	68	59	44	62	24	
Missing	35 (3.3%)	17	7	5	5	1	
**ER status**							0.52 (high)
Positive	800 (74.7%)	442	223	56	33	46	
Negative	269 (25.1%)	13	5	86	145	20	
Missing/Indeterminate	2 (0.2%)	1	0	0	0	1	
**PgR status**							0.45 (high)
Positive	670 (62.6%)	398	175	37	18	42	
Negative	397 (37.1%)	56	53	104	160	24	
Missing/Indeterminate	4 (0.4%)	2	0	1	0	1	
**HER2 status**							0.39 (high)
Positive	219 (20.4%)	45	33	107	15	19	
Negative	843 (78.7%)	407	191	35	163	47	
Missing/Equivocal	9 (0.8%)	4	4	0	0	1	
**Ki67 (%) - median=30, range=0-100**							
< = 20%	422 (39.4%)	284	54	38	19	27	0.32 (medium)
> 20%	607 (56.7%)	159	161	100	155	32	
Missing/Indeterminate	42 (3.9%)	13	13	4	4	8	

^#^The whole cohort distribution is shown as absolute number of patients and percentage of the total, while distribution according to subtypes are shown in number of patients. AOV: analysis of variance. For age at diagnosis among different PAM50 subtypes, categories with significant differences (Tukey HSD, alpha = 0.05) are indicated by a change on the superindex letter (i.e. a vs b are significantly different). For the remaining variables, the association between each of them and PAM50 subtypes was always significant (chi-squared test, p < 0.001 in all cases). Hue represents the sign of the standardized chi-squared residual; red hue indicates higher observed than expected counts, and blue indicates lower observed than expected counts. Color saturation represents the absolute value of the standardized chi-squared residuals; more saturation indicates a larger deviation from the expected counts. Low, medium and high refer to the strength of the association seen between subtypes and the clinicopathological characteristics. A Cramer’s V value of 0.2 or less indicates a weak association, between 0.2 and 0.3 a moderate association and higher than 0.3 a strong association.

### Comparative Prognostic Analysis of PAM50 and the IHC-Based Classifiers

**Figure 2:**
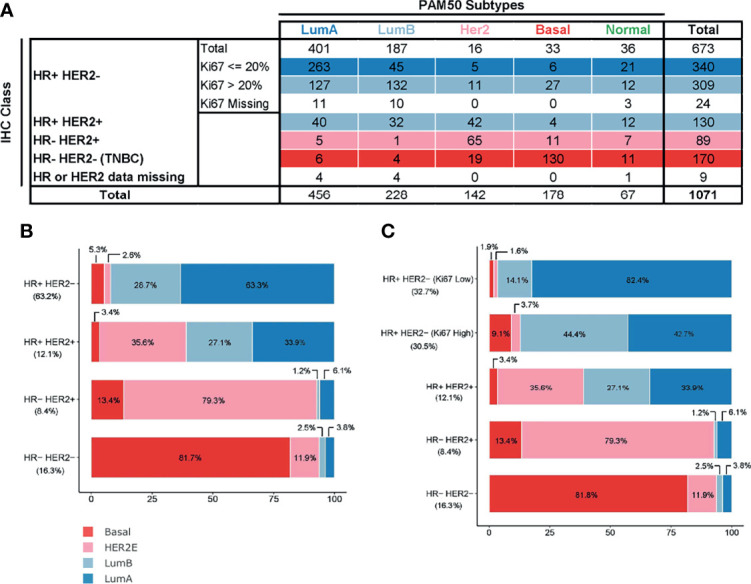
Comparison of PAM50 subtypes (excluding normal-like) and surrogate, immunohistochemistry-based subtypes present in MPBCS patients. **(A)** Frequency table for each category. Row colors represent the conventional correspondence between intrinsic and surrogate subtypes.**(B)** Classic IHC surrogate subtypes as defined by ER and PgR hormone receptors (HRs) and HER2 receptors (n = 996). **(C)** St Gallen IHC surrogate subtypes as defined by HR, HER2 receptors and high (more than 20%) or low (equal or less than 20%) levels of Ki67-positive cells. (n = 975). Patients with missing Ki67 values are excluded. Percentages in the x-axis and within each bar correspond to those of PAM50 subtypes in each surrogate group. Percentages in the y-axis correspond to the proportion of each surrogate subtype in the patients’ total. The total number of patients used for each panel excludes patients with missing values in any of the IHC determinations and those who were labeled as normal-like by PAM50.

IHC-based surrogate subtypes were calculated according to both classic IHC and a modification of the St Gallen 2013 criteria (St Gallen IHC). A total of 673/1062 samples (63.4%) were HR+/HER2-, of which 309 were classified as highly proliferative tumors (Ki67>20%) (**Figure 2A**). Of the remaining samples, 130 (12.2%) were HR+/HER2+, 89 (8.4%) were HR-/HER2+ and 170 (16.0%) were triple negative (TNBC) (**Figure 2A**).

Only ~60% of PAM50 assigned patients (622/995) could be classified into an equivalent IHC surrogate subtype (**Figure 2A**). The most consistent category was TNBC, which included 73.0% (130/178) of Basal cases (**Figures 2B, C**). The most heterogeneous subtype was the HR+ subtype, which included LumA, LumB and HER2E tumors in varying proportions (**Figure 2B, C**). HER2E tumors were distributed in almost equal proportions between HR+ and HR- classes (**Figures 2B, C**). The correlation between IHC and PAM50 subtypes was statistically significant (p < 0.001) with a Cramer’s V of 0.57 and of 0.61for classic IHC and St Gallen IHC, respectively (**Figures 2B, C**).

**Figure 3:**
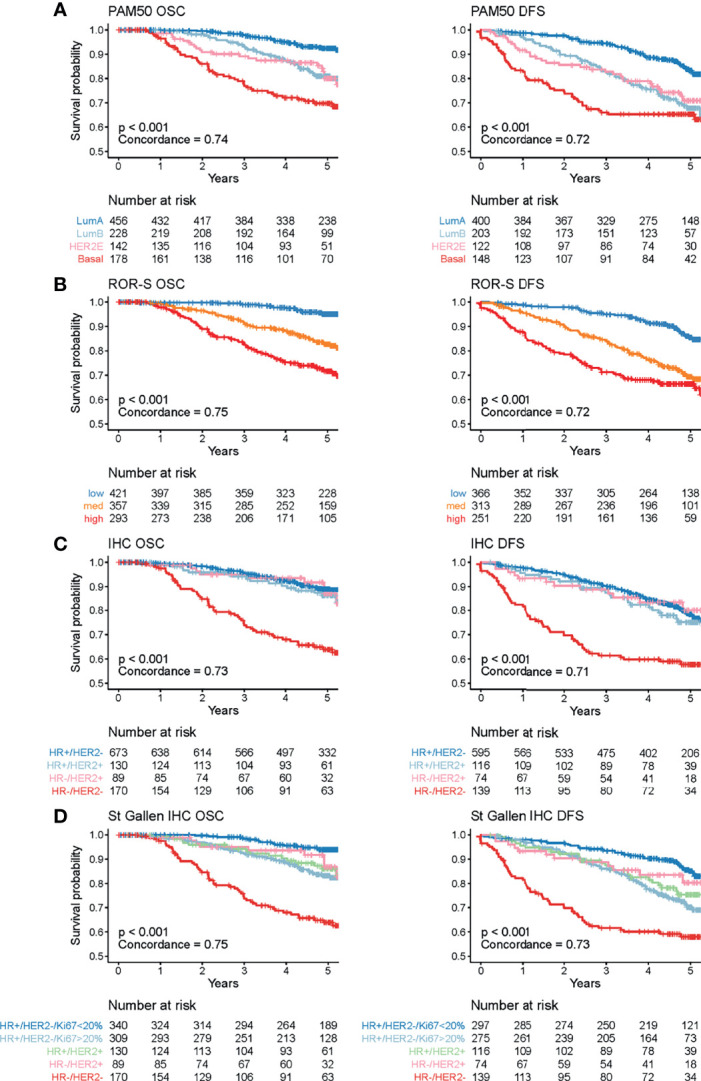
Cancer-related survival (OSC) and disease-free survival (DFS) of the MPBCS cohort according to different classifications. Each graph shows the Kaplan-Meier survival curves for each classification. **(A)** PAM50, **(B)** ROR-S, **(C)** classic IHC, **(D)** St Gallen IHC. Each class color is defined at the table of number at risk, below each graph. The p-value included in the graphs corresponds to the significance value of the log-rank test for all groups. Correlation (i.e., C-index) for each of the classifiers are also shown.

Survival curves according to the different classifications are shown in **Figure 3**, and their corresponding hazard ratios (HRs) against the class with the best survival are included in **Supplementary Table S2**. As all clinical and pathological variables showed associations with subtypes (**Table 1**), a univariate Cox model was selected for comparisons. Of note, multivariate analysis including age and clinical stage as covariables confirmed the prognostic value of subtypes (**Supplementary Table S2**)

PAM50 subtypes were prognostic for OSC and DFS (**Figure 3A**). Patients with LumA tumors had the best outcome, and those with Basal tumors had the worst outcome [HR 4.7 (confidence interval (CI) 3.0-7.5)]. Patients with eitherLumB [HR 2.5 (CI 1.6-4.1)] or HER2E [HR 2.7 (CI 1.6-4.7)] tumors showed similar intermediate survival. Consistent with that, ROR-S clearly discriminated patient survival into high [HR 6.5 (CI 3.9-10.7)], intermediate [HR 3.5 (CI 2.0-5.8)] and low (reference) risk groups (**Figure 3B**). The ROR-S obtained the highest correlation index for both OSC and DFS (**Figure 3**).

Classic IHC was only able to discriminate TNBC patient outcome from the rest (**Figure 3C** and **Supplementary Table S2**). However, when Ki67% was used to distinguish highly proliferative HR+ tumors (St Gallen IHC), the difference in survival between HR+HER2-high Ki67 cases [HR 2.8 (CI 1.6-4.8)] and HR+HER2-lowKi67 cases became apparent (**Figure 3D** and **Supplementary Table S2**).

### Enriched Pathways in the Intrinsic Subgroups of the MPBCS Cohort

**Figure 4:**
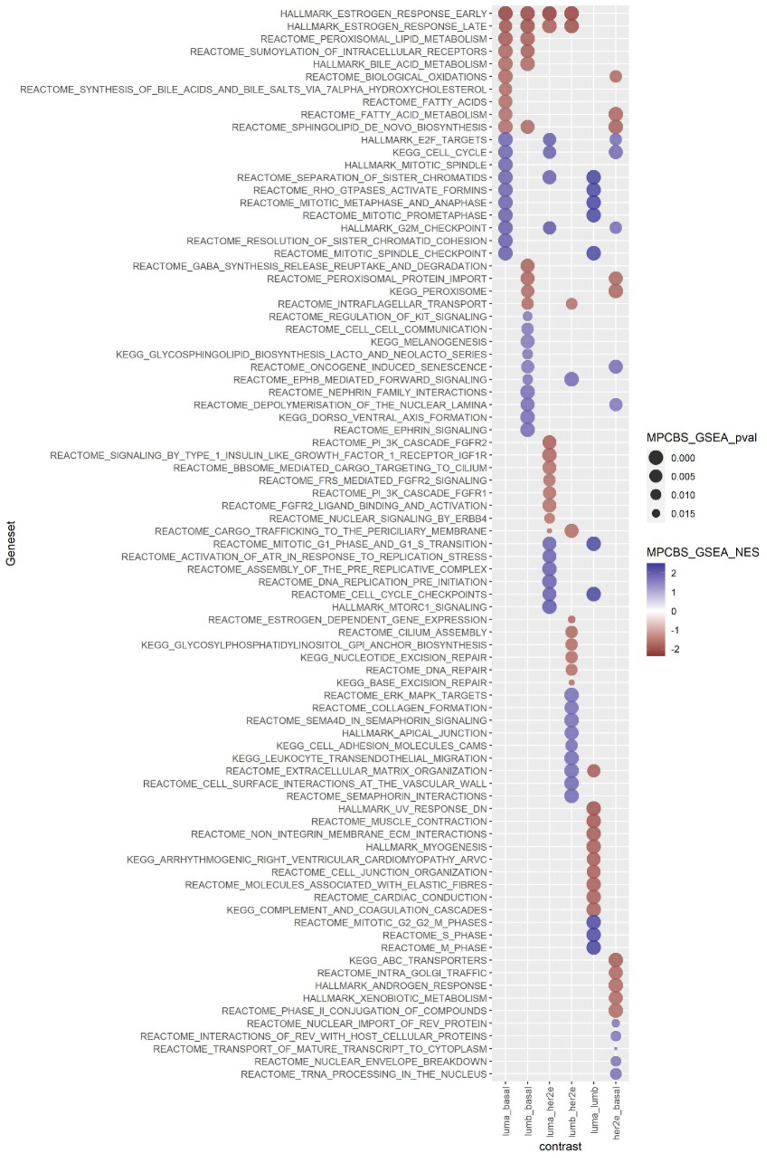
Summary of the top ten pathways enriched in each PAM50 subtype comparison according to GSEA, in the MPBCS cohort. Red and blue represent the enrichment of those terms in the first and second condition of each comparison, respectively; the color hue of circles indicate the NES magnitude and the size of the circle reflects the p-value of the enrichment.

For a deeper understanding of our Latin American cohort in molecular terms, we studied which biological pathways were enriched in each intrinsic subtype using different approaches. For enrichment analysis, we applied batch correction to the gene expression matrix, which accounted for a slight variation introduced by the year of array hybridization seen in the third PCA component (**Supplementary File 2** – Extended Methods). Both GSEA and GSVA showed a general concordance in significantly enriched pathways (p<0·05) in the comparisons among subtypes of the MPBCS cohort (**Supplementary Table S1**). As an example, **Figure 4** shows the top ten significant terms found enriched by GSEA in each comparison. **Table 2** summarizes the most relevant enriched pathways in GSEA and/or GSVA according to the condition in which they showed positive enrichment.

**Table 2:** Summary of top enriched gene sets by GSEA and GSVA (using all genes) in the MPBCS cohort.

	Enriched in first condition	Enriched in second condition
LumA vs Basal	Estrogen response	Cell cycle and mitosis (including TP53 activity)
	Peroxisoma/fatty acid metabolism	DNA replication
	Synthesis of glycosylphosphatidylinositol	MYC targets
	Biological oxidations	
LumB vs Basal	Estrogen response	Control of morphogenic processes
	Peroxisoma/fatty acid metabolism	Cell-cell communication
	Sumoylation of intracellular receptors	Signaling by EGFR
	Biological oxidations	
LumA vs HER2E	Estrogen response	Cell cycle and mitosis (including TP53 activity)
	Growth factor signaling (ERBB4, FGFR, ILGF, PI3K cascades)	DNA replication
		MYC targets
		PTEN regulation of stability and activity
LumB vs HER2E	Estrogen response	Extracellular matrix organization/collagen assembly
	DNA repair	Signaling by ERBB2
	Synthesis of glycosylphosphatidylinositol	Signaling by EGFR
		MAPK signaling pathway
		Cell adhesion and migration
LumA vs LumB	Extracellular matrix organization	Cell cycle and mitosis (including TP53 activity)
	Cell-cell and cell-matrix adhesion	DNA replication
	PI3K-AKT signaling in cancer	MYC targets
	MAPK signaling pathway	
	Myogenesis and muscle contraction	
HER2E vs Basal	Peroxisome/Fatty acid metabolism/steroid biosynthesis	Cell cycle and mitosis (including TP53 activity)
	Biological oxidations	MYC targets
	Xenobiotic metabolism	Intracellular transport
	Downregulation of ERBB2 signaling	
	Androgen response	

Even when all genes evaluated were used for these enrichment analyses, the pathways that stood out in every contrast included those represented by the PAM50 genes. Indeed, all luminal contrasts were enriched mainly on the ER pathway activation. Basal tumors exhibited enrichment of terms related to proliferation and DNA replication and repair (including *TP53*- and *MYC*-related pathways) in all contrasts but LumB. Cell proliferation was not enriched in the LumB vs Basal or in LumB vs HER2E comparisons indicating that LumB tumors in our cohort do not differ from other aggressive tumors in terms of proliferation capacity (**Figure 4** and **Table 2**). Enhanced proliferation in LumB tumors was also the most significant distinction between LumB and LumA tumors, confirming the association of LumB with high Ki67 levels (**Figure 4** and **Table 2**).

Terms associated with fatty acid metabolism and peroxidation stood out as characteristics of the luminal phenotype, as were significant both in the LumA vs Basal and LumB vs Basal comparisons (**Figure 4** and **Table 2**).

Terms related to *ERBB2* signaling appeared significantly enriched with respect to both LumA and LumB only on GSVA (**Table 2** and **Supplementary Table S1**). The LumA vs HER2E comparison showed that HER2E tumors were more proliferative, while LumA were enriched in different growth factor pathways including the PI3K cascade. The comparison between LumB and HER2E indicated an enrichment of DNA repair pathways in the LumB subtype and several terms related to extracellular matrix and cell adhesion enriched in HER2E. In general, both HER2E and Basal tumors showed upregulation of pathways associated with cell-cell communication when compared with luminal groups. The paired comparison between HER2E and Basal groups once again showed the strong activation of the cell cycle (also including the *TP53* and *MYC* pathways) seen in Basal tumors, while HER2E tumors showed enrichment of *ERBB2* and redox pathway terms in the GSVA analysis (**Supplementary Table S1**), as well as certain differential hormone dependency indicated by the regulation of androgen response (**Figure 4** and **Table 2**).

### Analysis of the Activity of Transcription Factors (TF) Between PAM50 Subtypes

**Figure 5:**
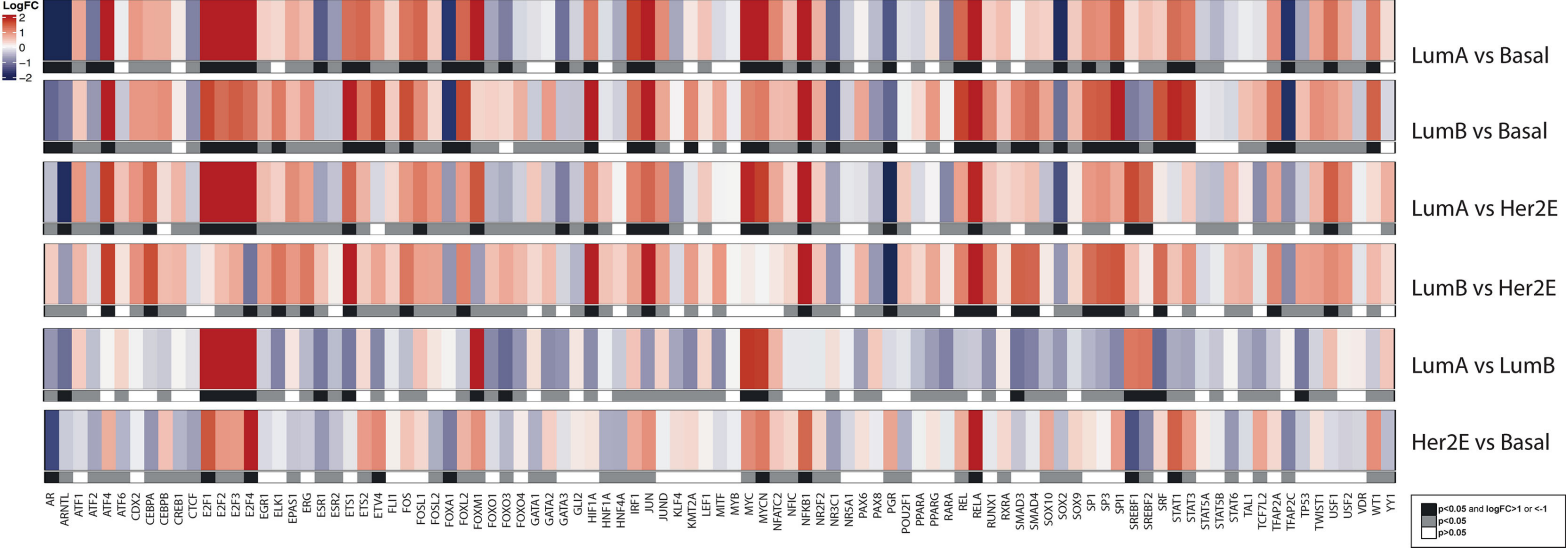
Differentially activated TF among PAM50 subtypes in the MPBCS cohort. The differential activities found for each TF (columns) in each PAM50 subtype contrast (rows) are shown in this heatmap. Z-scores are shown for the second term in the comparison (e.g. the red hue in the first row correspond to a TF activated in Basal samples). Below each row of the heatmap, a square in a gradient from white to black represent the p-value and logFC of the differential activity for each TF in each comparison; white correspond to non-significant comparisons.

To search specifically for TF networks differentially activated in each PAM50 subtype comparison, we analyzed the MPBCS expression matrix using DoRothEA and then search for differentially activated TF between each unpaired subtype comparison. Among the most differentially regulated, **Figure 5** shows the E2F and MYC families of proliferation-related TF more activated in Basal samples in all comparisons. The JUN/FOS transcription complex, essential for MAPK-associated proliferation, is also prevalent in the more agressive Basal and HER2E tumors. On the contrary, *PGR*, *ESR1* and *AR* transcriptional activities are enhanced in hormonal-driven LumA and LumB tumors. *FOXA1* and *GATA3* TF are both activated in luminal subtype, as expected, given that both *FOXA1* and *GATA3* are part of the luminal molecular signature. *NFKB1*, a master TF of aggresiveness and inflammation, and its counterpart *RELA* are highly activated in Basal and HER2E samples. STAT networks, involved in antigen presentation and immune response, are also activated in Basal samples, as well as *TWIST*, a TF associated to the epithelial-mesenchymal transition.

### Subtype-Specific Enriched Pathway Comparison Between MPBCS and TCGA

To further search for unique molecular characteristics among subtypes of the Latin American cohort, we performed the GSEA and GSVA contrasts between PAM50 subtypes with the publicly available data from TCGA and then compared the enriched pathways found in each cohort. For this comparison we selected only stage II-III TCGA breast cancer patients with PAM50 subtype assignment (TCGA*rf*) that rendered 19547 genes in common with our dataset. As expected, we observed a high degree of concordance between cohorts (**Supplementary Figure S2**). The NES for significantly enriched terms in MPBCS correlated in general with those seen for the same terms in TCGA*rf*, in all contrasts. Only a few isolated significant terms showed an opposite sign (**Supplementary Figure S2**, dots in red, term names within each graphic). The full list of TCGA enriched pathways and their NES scores with p-values is also included in **Supplementary Table S1**.

### Differential Analysis of Components of the Immune System

MetaCore pathway analysis of the two cohorts using DEGs showed additional significant information beyond those already found by the gene set enrichment analyses, such as the enrichment in cell-surface receptors associated with the crosstalk between the epithelial malignant cell compartment and the tumor stroma (e.g., IGF1-R, FGF3-R, HGF-R, IBP-4) in LumB tumors and the activation of Wnt signaling in HER2E, with overexpression of several members of the Wnt family and Frizzled. Of note, terms related to the most relevant innate and adaptive immune functions dominated in all Basal comparisons only when using DEGs exclusive of the MPBCS cohort and not among the TCGA DEGs (**Supplementary Table S3**). Indeed, among those MPBCS DEGs that were not found differentially expressed in the TCGA contrasts we could identify several genes encoding proteins involved in immunological synapses, such as the costimulatory CD40 (*CD40* gene), the immunosuppresive PD-L1 (*CD274* gene), and members of the signaling lymphocyte activation molecule (SLAM) family, which were elevated in Basal tumors compared to LumA tumors. In this comparison, genes related to T-cell and B-cell modulation such as IL-12, IL-21, TNFβ, and IFNγ were also overexpressed in Basal tumors, as well as several proinflammatory chemokines (e.g., GRO-1 and 2, CCL7, IL-6) and chemokine receptors (e.g., CCR5, CXCR3) associated with lymphocyte and neutrophil presence and/or activity. Furthermore, a family of histones identified by Metacore as relevant in the formation of neutrophil extracellular traps (NETs) were upregulated in proliferative tumors compared to LumA tumors, with their expression highest in LumB tumors.

**Figure 6:**
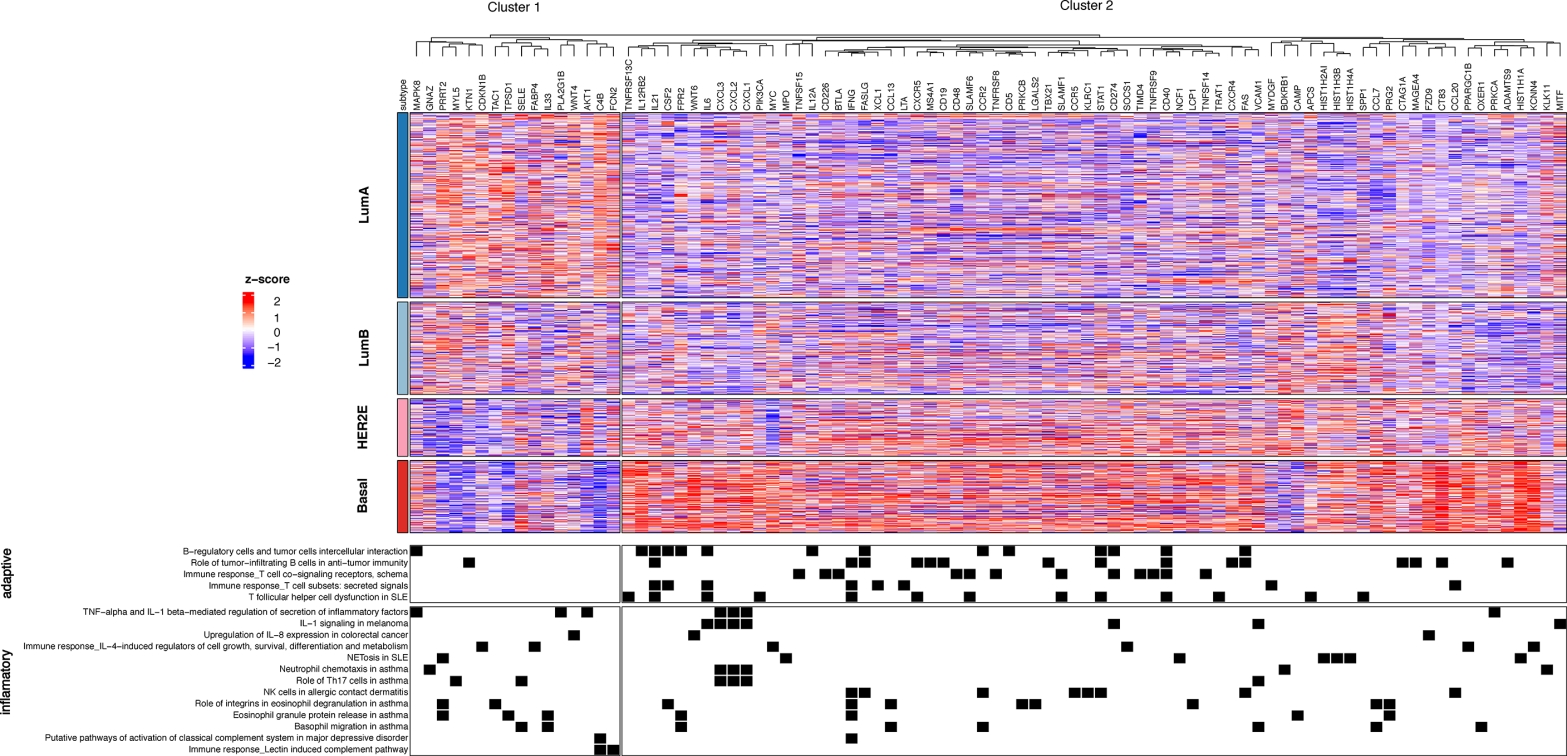
Clustering of differentially expressed genes related to immune terms in all PAM50 subtypes of the MPBCS cohort. The top panel shows the heatmap derived from a hierarchical clustering (k = 2); genes included in the enriched MetaCore pathways are in columns and tumors (grouped by PAM50 subtypes) are in rows. The bottom panel lists the immune-related enriched MetaCore pathways; black squares indicate the presence of a selected gene in any of the MetaCore terms.

As several of the MetaCore immune-related differential terms were found in more than one paired comparison, we selected only the MPBCS DEGs contained in those terms and used them for a hierarchical clustering (**Figure 6**). Two main clusters of genes become apparent, a smaller one with genes overexpressed in luminal samples (Cluster 1, **Figure 6**) and one with genes overexpressed in Basal and most HER2E samples (Cluster 2, **Figure 6**). Genes belonging to the adaptive immune system were almost exclusively in the second cluster with high expression in Basal and HER2E samples. On the other side, different set of genes involved in inflammatory pathways were overexpressed along all subtypes (Clusters 1 and 2, **Figure 6**). However, those with broader activities, that is, with important non-immune functions as well, were the ones enriched in LumA and LumB tumors (Cluster 1, **Figure 6**). Among them we found *PRRT2*, a tumor suppressor gene that inhibits proliferation, and genes involved in the DNA damage response and/or growth factor signaling cascades (e.g. *CDKN1B, MAPK8, AKT1* and *WNT4*). Of note, immune-related pathways were seldom observed among the top ten TCGA-exclusive differential terms of any contrast (**Supplementary Table S3**), although the presence of the adoptive immune system in TCGA and other breast cancer cohorts has been widely reported.

**Figure 7:**
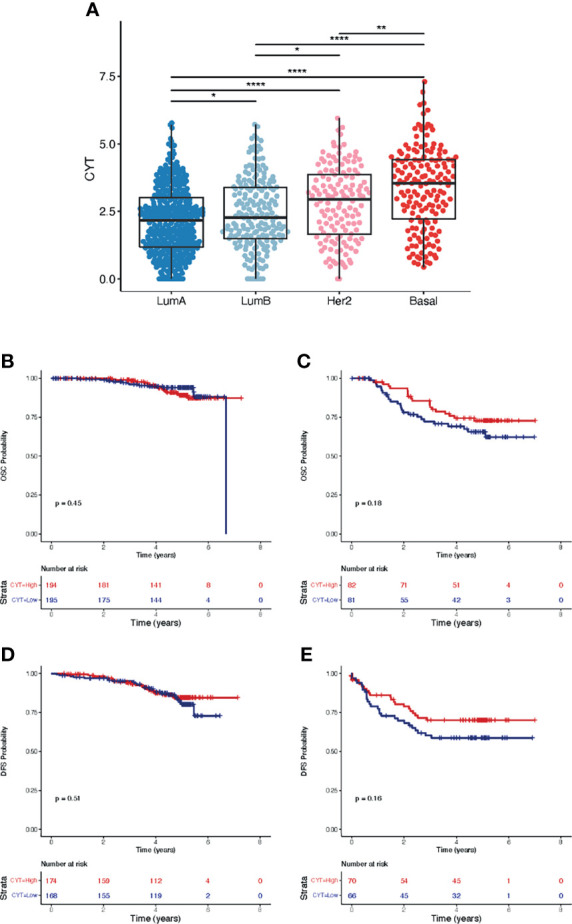
Cytolytic score CYT and survival analysis according to PAM50 subtypes in the MPBCS cohort. **(A)**, distribution of CYT among PAM50 subtypes. Medians were compared using the Kruskal-Wallis test (p= 2.2e-16). Adjusted p-values for the paired comparisons using Dunn’s test are defined as follows: *< 0.05, **< 0.01, ****< 0.0001. **(B, C)**, Kaplan-Meier curves and survival analysis for cancer-related overall survival (OSC) according to a binary assignation of high (i.e. above median) and low CYT to LumA **(B)** and Basal **(C)** tumors. **(D, E)**, Kaplan-Meier curves and survival analysis for disease-free survival (DFS) according to the same binary assignation of high (i.e. above median) and low CYT to LumA **(D)** and Basal **(E)** tumors.

The cytolytic score (CYT) has been used as indicator of the presence of tumor infiltrating lymphocytes (TILs). We calculated the CYT for all samples, and their distribution among the different subtypes is shown in **Figure 7A**. Significantly higher CYTs were seen in Basal samples, followed by HER2E samples. Luminal A and B samples have the lowest scores, although the shape of the boxplot may indicate a bimodal distribution in those samples. Next, we explored if tumors with high CYT (defined as CYTs above the median) showed differences in survival compared to those with low CYTs. At the level of the whole MPBCS cohort, survival analysis showed a slight tendency for better OSC and DFS in those patients with high CYTs, although the difference was not significant (not shown). When Basal and LumA samples were separately analyzed (with their own median value as threshold), CYT did not show OSC or DFS prognostic value in LumA samples (**Figure 7B, D**), while a tendency (still non-significant) for better prognosis with higher CYTs was seen for Basal samples (**Figures 7C, E**). The same by-subtype tendency but with worse p-values was seen using the whole cohort median as the threshold of low and high CYTs (not shown).

In recent years several molecular signatures exclusive for TNBC has been described, most of them converging in discrete molecular subtypes that differ in their expression of proliferation, immune and mesenchymal genes. We have applied Lehmann’s signature to classify the MPBCS TNBC cohort in the original 6 subgroups (TNBC-6) (25), in Lehmann’s refined TNBC-4 subtypes (26) and in Lehmann’s derived Bareche-2018 five-class groups (27). **Supplementary Figure S3** shows the relative distribution of subtypes within TNBC for each molecular signature (A), and their corresponding OSC (B) and DFS (C) survival curves. Confirming the results obtained with other cohorts, no significant differences could be found among any of the subtypes in any of the versions of the signatures (26).

## Discussion

The present study is the first multicountry, multicentric effort in Latin America, in which each participating institution generated a local biobank, performed molecular profiling, and registered its own clinical and transcriptional data by following strict consensus protocols and standard operating procedures. This resulted in a homogeneous dataset of high-quality, state-of-the-art clinical, pathological, and molecular data with minimum interinstitutional variation and a low percentage of missing data. This model of collaboration should be encouraged to lead to more diverse databases.

The clinicopathological and molecular features of the MPBCS cohort appear similar to those described in other hospital-based Latin American studies (8, 30–34). Non-LumA intrinsic subtypes accounted for more than half of the tumors in the MPBCS cohort, indicating the enrichment of this cohort in more aggressive molecular subtypes. However, compared with the LACE cohort, in which Sweeney et al. (8) analyzed the distribution of PAM50-derived subtypes in the Hispanic/Latino cases, the relative distribution of non-LumA subtypes within the MPBCS cohort slightly favored the Basal subtype over the HER2E cases and LumB cases (i.e., Basal/HER2E ratio 1.2 in MPBCS vs 0.7 in Hispanic LACE; Basal/LumB ratio 0.8 in MPBCS vs 0.5 in Hispanic LACE). Although this shift towards the Basal subtype most likely reflects the inclusion criteria of locally advanced stage (II/III) tumors (5, 35), we cannot disregard the fact that the diversity of our cohort might mask the effect of the ancestry in this distribution. Further work on the MPBCS will address this point.

For the first time, the 5-year prognostic ability of PAM50 could be establish in a large Latin American-based cohort. The prognostic power of PAM50 subtypes is compatible with what has been reported for node-negative and node-positive disease in other cohorts (17, 36–38). Albeit the prognostic power of IHC St Gallen for all the survival variables is acceptable and the C-indexes are comparable to those of PAM50, PAM50 still performs better at discriminating risk, as was already described for other cohorts (37, 39). Furthermore, PAM50-derived ROR-S (36) successfully classifies tumors into low, intermediate and high-risk groups with an even better performance than PAM50. Ongoing studies are focused in the exploration of other factors (40) and molecular signatures (37, 41) that can be used for further improving the prognostic value in this cohort.

Gene expression-based pathway analysis showed a general concordance of differentially expressed pathways between intrinsic subtypes with those previously described in the literature (42–45). GSEA/GSVA analyses prove that the major molecular characteristics of stage II and III breast cancer in patients from this Latin American cohort are similar to those of stage-matched TCGA patients. Proliferation, cell cycle control and DNA damage repair pathways were related to LumB, HER2E and Basal tumors, while LumA tumors showed a strong dependence on the estrogen pathway activation. Master TF activated in each contrast analysis were consistent with these main driver pathways, as already noted in the literature (42). Currently there is a general consensus in that each breast cancer intrinsic subtype represent an average phenotype resulting from an heterogeneous composition of cells within a tumor, and that the aggressiveness of a breast tumor is dictated by the different combination of cells present in the tumor (46–48). For this cohort, our multiple molecular analyses support this hypothesis and acknowledges the continuum of molecular phenotypes that coexists within each intrinsic subtype.

Among the findings that we consider worth to further explore, we observed upregulated fatty acid metabolic pathways in HR+ tumors. This finding, although already noted by the seminal work of Perou et al. (49) and others, can be reanalyzed in the light of recent studies that named fatty acid metabolism as a critical pathway related to cancer cell capacity to metastasize (50). The reprogramming of lipid metabolism is a hallmark of many cancers, including breast cancer (51). Our evidence points to the capacity of HR+ tumors for *de novo* fatty acid synthesis, mobilization and oxidation, in contrast to TNBC, which is more dependent on the uptake and storage of exogenous fatty acids (52). Further analysis will be done to explore the relationship between obesity, fatty acid metabolism and recurrence in luminal tumors, as the epidemiological and ancestral characteristics of the MPBCS cohort may bear subtler differences with other cohorts.

Interestingly, MetaCore analyses highlighted GO terms related to inflammatory/immune response that were only prominent in the MPBCS cohort and not in the TCGA samples; nevertheless, this is not a unique feature of MPBCS, as other more specific immunogenomic analysis of the TCGA database has already associated most Basal and some of HER2+ and LumB breast cancers with an IFN-γ dominant class, with a high content of CD8-T cells and of TCR diversity (53). The strong presence of the adaptive immune system in Basal or TNBC tumors has been extensively described in the literature and was linked to the presence of tumor-infiltrating lymphocytes (26, 27, 54–56). The score CYT described for our cohort is also consistent with these observations. Although underpowered, the tendency of high-CYT Basal (but not luminal) tumors to show better prognosis in the MPBCS cohort is consistent with previous evidence (57). Moreover, our evidence seems to be in line with the value of TIL content as a good prognostic marker in TNBC but not in luminal tumors (58, 59). These observations stress the importance of understanding the role of the immune response in the context of the different driving processes of tumor progression and therapy response in luminal and basal breast cancer.

CYT was also proposed as biomarker of response to immunotherapy in several tumors (60), although it is still not recognized as such in breast cancer (61). Another feature seen in our Basal samples, the expression of PD-L1, has been approved as biomarker for immune checkpoint blockade in TNBC (61, 62) although it turned out to be ineffective in certain settings (63). New prospective studies built under the example of the MPBCS might explore if the combination of both CYT and PD-L1 expression can be used as better predictive biomarkers of response to immunotherapy in Latin American TNBC patients.

Different TNBC classifications have been developed and their prognostic and predictive power studied (26, 27, 54, 64, 65). None of them have demonstrated a clear, robust prognostic value (26), although they have recently been shown as useful for predicting response to neoadjuvant chemotherapy in the BrighTNess trial (66). Our work proved that the classification of TNBC tumors into subtypes in the MPBCS cohort was feasible, although our study was not designed for detecting differences in survival or treatment response according to TNBC subtypes and it is inherently underpowered for these purposes.

Our TF analysis is consistent with the already observed *FOXA1*-driven immunosuppression in luminal samples (67) and the *NFKB1/STAT*-driven immune response in the Basal and, to a lesser extent, HER2E tumors (68). Other immune characteristics of our cohort are supported with less evidence in the literature but are worth to be studied further. For example, the literature suggest that B-cell markers in TNBC could be used as indicators of good prognosis (69) or that tumor-associated neutrophils (TANs) can be correlated with poor prognosis (70). In particular, neutrophil activity in NETs has been associated with sterile inflammation and tumor aggressiveness in breast cancer (71).

Interestingly, some recent reports addressed putative ancestral differences in the immune environment of TNBC tumors, with mixed results according to race (72, 73). The present immune-related findings highlight the quality of the MPBCS transcriptomic data and, along with the molecular ancestry assignation already achieved in this cohort, pave the way for deeper analyses in the search for better therapeutic strategies for Latin American patients.

This study has some limitations. As already mentioned, the hospital-based characteristic and the bias towards advanced stages of this cohort prevent the evaluation of the actual subtype distribution, as could be done in a population-based cohort. It is possible that some variations in prognostic power may be seen when by-country, by-treatment and/or by-ancestry stratification are done. Ongoing studies are based on the hypothesis that a fine tuning of the differential molecular pathways associated with tumor growth and aggressiveness may be found when stratified sample analysis by genetic ancestry is performed.

Our first studies on the high-quality data of the MPBCS cohort have given long-sought evidence that the main gene-expression features of breast cancer described for other cohorts are roughly conserved across Latin American patients and are informative on prognosis. Further analysis will explore potential country- and/or ancestry-specific differences in prognosis and treatment outcomes in this cohort.

## US-LACRN Collaborators

Juan Abarca, Hospital Barros Luco Trudeau, Santiago, Chile. Pamela Acevedo, Hospital San José, Santiago, Chile. Graciela Acosta, Hospital Municipal de Oncología María Curie, Buenos Aires, Argentina. Gissel Acosta, Hospital Universitario de Clinicas “Manuel Quintela”, Montevideo, Uruguay. Gabriela Acosta Haab, Hospital Municipal de Oncología María Curie, Buenos Aires, Argentina. Ana Lilian Acosta Silva, Universidad de Sonora, Hermosillo, México. Keyla Teresa Acosta Torres, Hospital General de Zona No. 2, IMSS, Hermosillo, Hermosillo, México. Marta Aghazarian, Instituto Nacional del Cáncer, Montevideo, Uruguay. Carola Aguayo, Instituto de Salud Pública, Santiago, Chile. Bernardo Aizen, Hospital Central de las Fuerzas Armadas, Montevideo, Uruguay. Gustavo Alarcon Lopez, Hospital Integral de la Mujer en el Estado de Sonora, Hermosillo, México. Liz Almeida, Instituto Nacional de Câncer, Rio de Janeiro, Brazi. Ana Alvarez, Instituto de Oncología Angel Roffo, Buenos Aires, Argentina. Viviane Andrade, Hospital de Câncer de Barretos, Barretos, Brazil. Wenceslao Angeles-Bueno, Hospital de Especialidades CMNO-IMSS, Guadalajara, Mexico. Roberto Arai, Instituto de Câncer de São Paulo, São Paulo, Brazil. Priscila Elvira Arambula Barreras, Universidad de Sonora, Hermosillo, México. Ma. Isabel Aramburo Rubio, Hospital General de Zona No. 2, IMSS, Hermosillo, Hermosillo, México. Estrellita Araus, Hospital Barros Luco Trudeau, Santiago, Chile. Gonzalo Ardao, Hospital Central de las Fuerzas Armadas, Montevideo, Uruguay. Lilia A. Arellano-Jimenez, Universidad de Guadalajara, Guadalajara, Mexico. Felipe Argandoña, Hospital San Borja Arriarán, Santiago, Chile. Claudia Arias, Hospital Municipal de Oncología María Curie, Buenos Aires, Argentina. Ricardo Armisen, Universidad de Chile, Santiago, Chile. Mauricio Aspee, Hospital Luis Tisne, Santiago, Chile. Rodrigo Assar, Universidad de Chile, Santiago, Chile. Itzel Reneé Astiazarán Rascón, Universidad de Sonora, Hermosillo, México. Sebastian Astorga, Hospital San Borja Arriarán, Santiago, Chile. Maxwell Aviles Rodriguez, Centro Estatal de Oncología, Hermosillo, México. Antônio Bailão Junior, Hospital de Câncer de Barretos, Barretos, Brazil. Adolfo E. Barragan-Curiel, O.P.D. Nuevo Hospital Civil de Guadalajara, Guadalajara, Mexico. Adelfo Barragan-Ruiz, Hospital de Gineco-Obstricia CMNO-IMSS, Guadalajara, Mexico. Fernanda Bermudez, Programa Nacional para el Control del Cancer, Montevideo, Uruguay. Julia Bernachin, Hospital Universitario de Clinicas "Manuel Quintela", Montevideo, Uruguay. Wilfrido Bernal Herrera, Centro Estatal de Oncología, Hermosillo, México. Mara Bonet, Instituto de Oncología Angel Roffo, Buenos Aires, Argentina. Sarah Brnich, Fundación Instituto Leloir-CONICET, Buenos Aires, Argentina. Claudio Bustamante, Hospital San José, Santiago, Chile. Miguel Angel Bustamante, Hospital San Borja Arriarán, Santiago, Chile. Julio Bustos-Gomez, O.P.D. Nuevo Hospital Civil de Guadalajara, Guadalajara, Mexico. Felipe de J Bustos-Rodriguez, O.P.D. Nuevo Hospital Civil de Guadalajara, Guadalajara, Mexico. Janett Caballero Jasso, Hospital General de Zona No. 14, IMSS Hermosillo, Hermosillo, México. Angie Calfuman, Hospital Luis Tisne, Santiago, Chile. Antonio Hugo José Froes Marques, Campos, AC Camargo Cancer Center, São Paulo, Brazil. Mónica Campos, Hospital San Borja Arriarán, Santiago, Chile. Soledad Cano, Instituto Nacional del Cáncer, Santiago, Chile. Juan C. Canton-Romero, Hospital de Gineco-Obstricia CMNO-IMSS, Guadalajara, Mexico. Ricardo Cappetta, Hospital Municipal Diego Thompson, Buenos Aires, Argentina. Paulina Carmona, Grupo Oncológico Cooperativo Chileno de Investigación, Santiago, Chile. Fernando Carrizo, Instituto de Oncología Angel Roffo, Buenos Aires, Argentina. André Lopes Carvalho, Hospital de Câncer de Barretos, Barretos, Brazil. Erika Carvallo, Hospital San Borja Arriarán, Santiago, Chile. Julio Carzoglio, Instituto Nacional del Cáncer, Montevideo, Uruguay. Monica Casalnuovo, Hospital Municipal de Oncología María Curie, Buenos Aires, Argentina. Benedicta Caserta, Centro Hospitalario Pereira Rossell, Montevideo, Uruguay. Alvaro Castillo, Hospital San José, Santiago, Chile. César Castillo, Hospital Barros Luco Trudeau, Santiago, Chile. Roger Chammas, Instituto de Câncer de São Paulo, São Paulo, Brazil. Mario Alberto Chavez Zamudio, Hospital General Regional No. 1, IMSS, Obregon, Obregón, México. Loreto Chia, Hospital San José, Santiago, Chile. Elisa Chiarello, Hospital Municipal Diego Thompson, Buenos Aires, Argentina. Daniela Chirico, Fundación Instituto Leloir-CONICET, Buenos Aires, Argentina. Esther Cisneros-Quirarter, OPD Hospital Civil de Guadalajara, Universidad de Guadalajara, Guadalajara, Mexico. Minor Raul Cordero Bautista, Hospital General del Estado de Sonora, Hermosillo, México. Valeria Cornejo, Hospital San Borja Arriarán, Santiago, Chile. Baldemar Corral Villegas, Centro Estatal de Oncología, Hermosillo, México. Andrés Cortés, Grupo Oncológico Cooperativo Chileno de Investigación, Santiago, Chile. Sandra Cortés, Pontificia Universidad Católica de Chile, Santiago, Chile. Laura Cortes-Sanabria, Hospital de Especialidades CMNO-IMSS, Guadalajara, Mexico. German Salvador Cortez Zamorano, Universidad de Sonora, Hermosillo, México. Alejandro Corvalan, Grupo Oncológico Cooperativo Chileno de Investigación, Santiago, Chile. Adolfo Cruz, Hospital Barros Luco Trudeau, Santiago, Chile. Alba d'Aurora, Hospital San Borja Arriarán, Santiago, Chile. Sandra De la Fuente, Universidad de Chile, Santiago, Chile. Soledad De la Peña, Centro Hospitalario Pereira Rossell, Montevideo, Uruguay. Roberto de Leon Caballero, Hospital General del Estado de Sonora, Hermosillo, México. Mirian de Souza, Instituto Nacional de Câncer, Rio de Janeiro, Brazil. César Del Castillo, Hospital San Borja Arriarán, Santiago, Chile. Azucena Del-Toro-Valero, Universidad de Guadalajara, Guadalajara, Mexico. Mirtha Di Pretoro, Instituto de Oncología Angel Roffo, Buenos Aires, Argentina. Andrea Digonzelli, Hospital Regional de Agudos Eva Perón, Buenos Aires, Argentina. Jose El Ters, Instituto Nacional del Cáncer, Montevideo, Uruguay. Paula Escobar, Hospital Luis Tisne, Santiago, Chile. Marcela Estolaza, Hospital Luis Tisne, Santiago, Chile. Adriane Feijo Evangelista, Hospital de Câncer de Barretos, Barretos, Brazil. Marcelo Fanelli, AC Camargo Cancer Center, São Paulo, Brazil. Paulo Farias, Instituto Nacional de Câncer, Rio de Janeiro, Brazil. Graciela Fernandez, Hospital Central de las Fuerzas Armadas, Montevideo, Uruguay. José R. Filassi, Cancer Institute of São Paulo (ICESP), Sao Paulo University Medical School, Sao Paulo, Brazil. Natalia Filgueiras, Hospital Municipal de Oncología María Curie, Buenos Aires, Argentina. Diego Flaks, Hospital Municipal de Oncología María Curie, Buenos Aires, Argentina. Edgar G. Flores-Ayala, Instituto Jalisciense de Cancerologia, Guadalajara, Mexico. Maria R. Flores-Marquez, Hospital de Especialidades CMNO-IMSS, Guadalajara, Mexico. David Franco Hughes, Universidad de Sonora, Hermosillo, México. Karina Franco-Topete, O.P.D. Nuevo Hospital Civil de Guadalajara, Guadalajara, Mexico. Jimena Freire, Instituto Nacional del Cáncer, Montevideo, Uruguay. Cristobal Fresno, Universidad Católica de Córdoba, Córdoba, Argentina. Romina Gabrielli, Centro Hospitalario Pereira Rossell, Montevideo, Uruguay. Mario Gallegos, Hospital San Borja Arriarán, Santiago, Chile. Jorge Gamboa, Hospital San Borja Arriarán, Santiago, Chile. Daiana Ganiewich, Fundación Instituto Leloir-CONICET, Buenos Aires, Argentina. Carlos Garbovesky, Hospital Municipal de Oncología María Curie, Buenos Aires, Argentina. Ruben Alejandro Garcia Munguia, Hospital de Ginecopediatría, IMSS, Hermosillo, Hermosillo, México. Ricardo Garcia-Gaeta, Universidad de Guadalajara, Guadalajara, Mexico. Alma C. Garcia-Martinez, Universidad de Guadalajara, Guadalajara, Mexico. Liliana Gimenez, Instituto de Oncología Angel Roffo, Buenos Aires, Argentina. Hector Gómez Silveira, Hospital Municipal Diego Thompson, Buenos Aires, Argentina. Mariana M. Gomez-Del Toro, Universidad de Guadalajara, Guadalajara, Mexico. Marcela Gonzalez, Hospital Regional de Agudos Eva Perón, Buenos Aires, Argentina. Alicia Gonzalez, Hospital Universitario de Clinicas “Manuel Quintela”, Montevideo, Uruguay. Germán González, Universidad Católica de Córdoba, Córdoba, Argentina. Cesar Osbaldo Gonzalez Mondaca, Hospital General de Zona No. 2, IMSS, Hermosillo, Hermosillo, México. Leivy P. Gonzalez-Ramirez, Universidad de Guadalajara, Guadalajara, Mexico. Beatriz Gonzalez-Ulloa, Hospital de Especialidades CMNO-IMSS, Guadalajara, Mexico. Susana Gorostidy, Instituto de Oncología Angel Roffo, Buenos Aires, Argentina. Mariela Grass, Instituto Nacional del Cáncer, Montevideo, Uruguay. Alfonso G. Guevara Torres, Centro Estatal de Oncología, Hermosillo, México. Lorena Gutierrez, Hospital San Borja Arriarán, Santiago, Chile. Susan A, Gutierrez-Rubio, Universidad de Guadalajara, Guadalajara, Mexico. Adrián Hannois, Hospital Regional de Agudos Eva Perón, Buenos Aires, Argentina. Andrew Hart, Universidad de Chile, Santiago, Chile. Steffen Härtel, Universidad de Chile, Santiago, Chile. Marcos Henriquez, Hospital Barros Luco Trudeau, Santiago, Chile. Rafael Hernandez Guevara, Hospital General Regional No. 1, IMSS, Obregon, Obregón, México. Miriam E. Hernandez-Franco, Universidad de Guadalajara, Guadalajara, Mexico. Manuel I. Herrera-Miramontes, Universidad de Guadalajara, Guadalajara, Mexico. Graciela Horton, Hospital Municipal de Oncología María Curie, Buenos Aires, Argentina. Gladys Ibañez, Hospital San José, Santiago, Chile. Martín Ipiña, Instituto de Oncología Angel Roffo, Buenos Aires, Argentina. Beatriz Jalfin, Hospital Regional de Agudos Eva Perón, Buenos Aires, Argentina. Lilian Jara, Universidad de Chile, Santiago, Chile. Raul Jara, Hospital Luis Tisne, Santiago, Chile. Maria Luisa Jaramillo, Hospital San Borja Arriarán, Santiago, Chile. David Javalera-Castro, Universidad de Guadalajara, Guadalajara, Mexico. Maria Eugenia Jimenez, Hospital Barros Luco Trudeau, Santiago, Chile. Hugo Ju, Grupo Oncológico Cooperativo Chileno de Investigación, Santiago, Chile. Nazareth Juárez Rusjan, Instituto de Oncología Angel Roffo, Buenos Aires, Argentina. Karen Juneman, Hospital Luis Tisne, Santiago, Chile. Ligia Maria Kerr, Hospital de Câncer de Barretos, Barretos, Brazil. Alejandra Krupelis, Hospital Municipal de Oncología María Curie, Buenos Aires, Argentina. Flor Esmeralda Larios-Jimenez, Universidad de Guadalajara, Guadalajara, Mexico. Jose Domingo Latorre, Hospital Municipal Diego Thompson, Buenos Aires, Argentina. Guillermo Laviña, Hospital Universitario de Clinicas “Manuel Quintela”, Montevideo, Uruguay. Fernando Lavista, Hospital Central de las Fuerzas Armadas, Montevideo, Uruguay. Irma Leticia León Duarte, Hospital General del Estado de Sonora, Hermosillo, México. Alberto Lescano, Hospital Municipal de Oncología María Curie, Buenos Aires, Argentina. Verónica Lezano, Grupo Oncológico Cooperativo Chileno de Investigación, Santiago, Chile. Rossana Mendoza Lopez, Instituto de Câncer de São Paulo, São Paulo, Brazil. Jose Guillermo Lopez Cervantes, Universidad de Sonora, Hermosillo, México. Miguel Enrique Lopez Muñoz, Universidad de Sonora, Hermosillo, México. Dora Loria, Instituto de Oncología Angel Roffo, Buenos Aires, Argentina. Alejandra Luque, Hospital Central de las Fuerzas Armadas, Montevideo, Uruguay. Alejandro Maass, Universidad de Chile, Santiago, Chile. Maria do Socorro Maciel , AC Camargo Cancer Center, São Paulo, Brazil. Flavia Rotea Mangone, Instituto de Câncer de São Paulo, São Paulo, Brazil. Jorge Mansilla, Universidad de Chile, Santiago, Chile. Katherine Marcelain, Universidad de Chile, Santiago, Chile. Carolina Mariani, Grupo Oncológico Cooperativo Chileno de Investigación, Santiago, Chile. Marcia Maria Chiquitelli Marques, Hospital de Câncer de Barretos, Barretos, Brazil. Reyna J. Martinez-Arriaga, Universidad de Guadalajara, Guadalajara, Mexico. Hector R. Martinez-Ramirez, Hospital de Especialidades CMNO-IMSS, Guadalajara, Mexico. Marcela Martins, Instituto de Câncer de São Paulo, São Paulo, Brazil. Alma G. Maya-Gonzalez, Universidad de Guadalajara, Guadalajara, Mexico. Brenda Mazzaferri, Hospital Municipal Diego Thompson, Buenos Aires, Argentina. Yadira Medina-Mora, Universidad de Guadalajara, Guadalajara, Mexico. Mariana Menini, Hospital Central de las Fuerzas Armadas, Montevideo, Uruguay. Silvia Míguez, Hospital Municipal de Oncología María Curie, Buenos Aires, Argentina. Soledad Milans, Hospital Universitario de Clinicas “Manuel Quintela”, Instituto Nacional del Cancer, Montevideo, Uruguay. Soledad Montes, Instituto Nacional del Cáncer, Santiago, Chile. Ana Verónica Morales Hernández, Universidad de Sonora, Hermosillo, México. Giberto Morga-Villela, Hospital de Especialidades CMNO-IMSS, Guadalajara, Mexico. Carla Morong, Hospital San Borja Arriarán, Santiago, Chile. Homero Muñoz, Programa Nacional para el Control del Cancer, Montevideo, Uruguay. Ignacio Miguel Muse, Programa Nacional para el Control del Cancer, Montevideo, Uruguay. Carina Mussetti, Centro Hospitalario Pereira Rossell, Montevideo, Uruguay. Eduardo Mussetti, Centro Hospitalario Pereira Rossell, Montevideo, Uruguay. Luis J. Najar-Acosta, Universidad de Guadalajara, Guadalajara, Mexico. Elisa Napolitano e Ferreira, AC Camargo Cancer Center, São Paulo, Brazil. Nancy E. Navarro-Ruiz, Universidad de Guadalajara, Guadalajara, Mexico. Cristina Noblía, Instituto de Oncología Angel Roffo, Buenos Aires, Argentina. João Soares Nunes, Hospital de Câncer de Barretos, Barretos, Brazil. Daniela Núñez, Hospital Regional de Agudos Eva Perón, Buenos Aires, Argentina. Fabiola Núñez, Hospital Luis Tisne, Santiago, Chile. Nilton Onari, Hospital de Câncer de Barretos, Barretos, Brazil. Emma M. Oropeza-De Anda, Universidad de Guadalajara, Guadalajara, Mexico. David Ortega Tirado, Universidad de Sonora, Hermosillo, México. Cynthia Aparecida Bueno de Toledo Osório, AC Camargo Cancer Center, São Paulo, Brazil. Carlos Eduardo Paiva, Hospital de Câncer de Barretos, Barretos, Brazil. Paulina Peñaloza, Hospital Luis Tisne, Santiago, Chile. Miguel Peredo-Navarro, Hospital de Especialidades CMNO-IMSS, Guadalajara, Mexico. David Pereira, Instituto de Oncología Angel Roffo, Buenos Aires, Argentina. Laura Perez Michel, Hospital General Regional No. 1, IMSS, Obregon, Obregón, México. Francisca Pino, Hospital Barros Luco Trudeau, Santiago, Chile. Tania Pino, Hospital San José, Santiago, Chile. Natalia Pinto, Hospital Luis Tisne, Santiago, Chile. Jessica Pizarro , Hospital Barros Luco Trudeau, Santiago, Chile. Carlos Pressa, Hospital Universitario de Clinicas “Manuel Quintela”, Montevideo, Uruguay. Enrique Ramirez, Hospital Municipal de Oncología María Curie, Buenos Aires, Argentina. Gladys E. Ramirez-Rosales, Universidad de Guadalajara, Guadalajara, Mexico. Claudia Ramis, Hospital San José, Santiago, Chile. Maritza Ramos-Ramirez, Universidad de Guadalajara, Guadalajara, Mexico. Adela Rascon Alcantar, Hospital Infantil del Estado de Sonora, Hermosillo, México. Silvana Ravaglio, Hospital Central de las Fuerzas Armadas, Montevideo, Uruguay. Francois Richard, Grupo Oncológico Cooperativo Chileno de Investigación, Santiago, Chile. Omar Rios Méndez, Hospital General del Estado de Sonora, Hermosillo, México. Ramón Enrique Robles Zepeda, Universidad de Sonora, Hermosillo, México. Iara Santana Rocha, Hospital de Câncer de Barretos, Barretos, Brazil. Natalia Rodriguez, Hospital Barros Luco Trudeau, Santiago, Chile. Vilma Rodriguez, Hospital Barros Luco Trudeau, Santiago, Chile. Maria Teresa Rodriguez, Hospital San José, Santiago, Chile. Diego Rodriguez-Gonzalez, Universidad de Guadalajara, Guadalajara, Mexico. Rosemeire A Roela, Instituto de Câncer de São Paulo, São Paulo, Brazil. Ana M Romero-Gomez, Universidad de Guadalajara, Guadalajara, Mexico. Ana M Rosales-Sandoval, Hospital de Especialidades CMNO-IMSS, Guadalajara, MexicoLidia A Rubio-Chavez, Universidad de Guadalajara, Guadalajara, Mexico. Omar V Rubio-Plascencia, Universidad de Guadalajara, Guadalajara, Mexico. Florencia Russo, Hospital Regional de Agudos Eva Perón, Buenos Aires, Argentina. Gaciela Sabini, Programa Nacional para el Control del Cancer, Montevideo, Uruguay. Isabel Saffie, Hospital Luis Tisne, Santiago, Chile. Helena Salina Dias, Cancer Institute of São Paulo (ICESP), Sao Paulo University Medical School, Sao Paulo, Brazil. Brenda Guadalupe Samaniego Soto, Universidad de Sonora, Hermosillo, México. Julio San Martino, Hospital Municipal Diego Thompson, Buenos Aires, Argentina. Benito Sanchez-Llamas, Hospital de Gineco-Obstricia CMNO-IMSS, Guadalajara, Mexico. Daniel Sat-Muñoz, Hospital de Gineco-Obstricia CMNO-IMSS, Guadalajara, Mexico. Mariana Savignano, Instituto de Oncología Angel Roffo, Buenos Aires, Argentina. Cristovam Scapulatempo Neto, Hospital de Câncer de Barretos, Barretos, Brazil. Max Mano Senna, Instituto de Câncer de São Paulo, São Paulo, Brazil. Carolina Silva, Hospital San Borja Arriarán, Santiago, Chile. Jaime Silvera, Centro Hospitalario Pereira Rossell, Montevideo, Uruguay. Isabele Small, Instituto Nacional de Câncer, Rio de Janeiro, Brazil. Fernando Soares, AC Camargo Cancer Center, São Paulo, Brazil. Iberê Soares, Instituto de Câncer de São Paulo, São Paulo, Brazil. Silvana Soares dos Santos, AC Camargo Cancer Center, São Paulo, Brazil. Evandro Sobrosa de Mello, Instituto de Câncer de São Paulo, São Paulo, Brazil. José Antonio Sola, Instituto Nacional del Cáncer, Santiago, Chile. Irene Sorín, Hospital Regional de Agudos Eva Perón, Buenos Aires, Argentina. Alejandra Sosa, Programa Nacional para el Control del Cancer, Montevideo, Uruguay. Anabella Sosa, Hospital Regional de Agudos Eva Perón, Buenos Aires, Argentina. Claudio Sosa, Centro Hospitalario Pereira Rossell, Montevideo, Uruguay. Sandra Soto, Instituto Nacional del Cancer, Montevideo, Uruguay. Cristiano de Pádua Souza, Hospital de Câncer de Barretos, Barretos, Brazil. Lucía Spangenberg, Institut Pasteur de Montevideo, Montevideo, Uruguay. Gustavo Steffanof, Instituto Nacional de Câncer, Rio de Janeiro, Brazil. Florencia Straminsky, Fundación Instituto Leloir-CONICET, Buenos Aires, Argentina. Mónica Tapia, Hospital Luis Tisne, Santiago, Chile. Raziel O. Tapia-Llanos, Universidad de Guadalajara, Guadalajara, Mexico. Geronimo M. Tavares-Macias, Hospital de Especialidades CMNO-IMSS, Guadalajara, Mexico. Guillermo Temperley, Hospital Municipal de Oncología María Curie, Buenos Aires, Argentina. Veronica Terzieff, Centro Hospitalario Pereira Rossell, Montevideo, Uruguay. Vicente Teti, Hospital Municipal de Oncología María Curie, Buenos Aires, Argentina. Javier Tognarelli, Instituto de Salud Pública, Santiago, Chile. Verónica Toledo, Hospital Luis Tisne, Santiago, Chile. Paulina Toro, Hospital Luis Tisne, Santiago, Chile. Roberto Torres, Instituto Nacional del Cáncer, Santiago, Chile. Mariana Torres-Palomares, Universidad de Guadalajara, Guadalajara, Mexico. Rogelio Troyo-San Roman, Universidad de Guadalajara, Guadalajara, Mexico. Hernan Urbano, Hospital Barros Luco Trudeau, Santiago, Chile. Nicolas Vacca, Programa Nacional para el Control del Cancer, Montevideo, Uruguay. Daniel Vaimberg, Hospital Regional de Agudos Eva Perón, Buenos Aires, Argentina. María Lourdes Valencia Peña, Universidad de Sonora, Hermosillo, México. Maria Lujan Vaselevich, Hospital Municipal Diego Thompson, Buenos Aires, Argentina. Jaime Vazquez-Nares, Instituto Jalisciense de Cancerología, Guadalajara, Mexico. Ezequiel Velez-Gomez, O.P.D. Nuevo Hospital Civil de Guadalajara, Guadalajara, Mexico. Laura N. Venegas-Godinez, Universidad de Guadalajara, Guadalajara, Mexico. Patricia Vercelli, Hospital Regional de Agudos Eva Perón, Buenos Aires, Argentina. Ricardo Verdugo, Universidad de Chile, Santiago, Chile. René Aloisio da Costa Vieira, Hospital de Câncer de Barretos, Barretos, Brazil. Marta Vilensky, Instituto de Oncología Angel Roffo, Buenos Aires, Argentina. María José Villarubias, Hospital Regional de Agudos Eva Perón, Buenos Aires, Argentina. Manuel Isaac Villegas Gómez, Universidad de Sonora, Hermosillo, México. Stella Viña, Instituto de Oncología Angel Roffo, Buenos Aires, Argentina. Silvia Vornetti, Hospital Municipal de Oncología María Curie, Buenos Aires, Argentina. Anapaula Hidemi Uema Watanabe, Hospital de Câncer de Barretos, Barretos, Brazil. Carlos Zamorano, Hospital Barros Luco Trudeau, Santiago, Chile. Luis Zapata, Hospital Barros Luco Trudeau, Santiago, Chile. Zdenka Zlatar, Grupo Oncológico Cooperativo Chileno de Investigación, Santiago.

## Data Availability Statement

The data that support the findings of this study are available from US-LACRN but restrictions apply to the availability of these data. Data are however available from the authors upon reasonable request and with permission of US-LACRN.

## Ethics Statement

The studies involving human participants were reviewed and approved by National Cancer Institute Ethics Committee (IRB #: 15CN055; iRIS Reference #: 549650) and the local institutional committees in each country. The patients provided their written informed consent to participate in this study.

## Author Contributions

Conceptualization and design, JG, OP, EA, AL, NA, AD-N, BM, CV, IA, RB, AB, JF, WF, FG, MG, LS, NC, DC, MC, SCa, ACa, SCr, WF, FG, MG, MN, RMR, RR, LD, MD-B, MH, VV, and TG. Supervision, OP, AL, NA, AD-N, BM, CV, EBA, IA, AB, JF, NC, DC, MC, SCa, ACa, SCr, WF, FG, MG, MN, RMR, RR, JC-C, LS, ACo, RD-C, RF-T, AM-M, AO-V, JR, CR, ES-G, AS-G, LZ, AD-A, AQ-R, LD, MD-B, JG, TG, MH, VV, OV, MO-M, ER-C, MCe, AG-E, EAF, and EA. Data collection, AL, NA, AD-N, BM, CV, EBA, IA, RB, JF, AB, JS, NC, DC, MC, SCa, ACa, SCr, WF, FG, MG, MN, RMR, RR, LS, JC-C, ACo, RD-C, RF-T, CG, SM, AM-M, AO-V, JR, CR, VS, ES-G, AS-G, AT, LZ, AD-A, AQ-R, MO-M, JQ, ER-C, MCe, AL-V, EAF, GG, and EA. Bioinformatic and statistical analysis, DR, JS, MCe, RB, EA, EAF, GG, ML-M, DA, and AL. Visualization, DR, JS, RB, EAF, and AL. Data interpretation, DR, JS, RB, EAF, LF, NA, OP, EA, JF, MCe, AD-A, AQ-R, and AL. Writing, DR, AL, EA, and OP. Reviewing and editing, LF, OP, AL, NA, AD-N, BM, CV, JF, EBA, IA, AB, DR, NC, DC, MC, SCa, ACa, SCr, WF, FG, MG, MN, RMR, JC-C, RR, LS, ACo, RD-C, RF-T, AM-M, AO-V, JR, CR, ES-G, AS-G, LZ, AD-A, AQ-R, LD, MD-B, JG, TG, MH, VV, OV, JQ, JS, MCe, EAF, AG-E, GG, AL-V, and EA. Funding acquisition, JG, TG, VV, MH, BM, NA, EA, AD-N, CV, MD-B, AL, DR, JS, AL, EA, and OP have verified the underlying data. All authors contributed to the article and approved the submitted version.

## Funding

Center for Global Health at the United States - National Cancer Institute at the National Institutes of Health (CGH-US(NCI), (contract award # HHSN2612010000871/NO2-PC-2010-00087), Fogarty International Center, Susan G. Komen for the Cure, Argentina, Fundación Argentina de Nanotecnología at the Ministry of Science, Technology, and Innovation, Agencia Nacional de Promoción Científica y Tecnológica, Instituto Nacional del Cáncer at the Ministry of Health, Brazil, Ministry of Health, Chile, Public Health Institute at the Ministry of Health, Mexico, Consejo Estatal de Ciencia y Tecnología de Jalisco (COECYTJAL) (no grant number applies, Universidad de Sonora. The CGH-NCI(US) had a role in study design and data curation. The rest of the sponsors have no role in the study design, nor in the collection, analysis, and interpretation of data, the writing of the report and the decision to submit the paper for publication.

## Conflict of Interest

The authors declare that the research was conducted in the absence of any commercial or financial relationships that could be construed as a potential conflict of interest.

## Publisher’s Note

All claims expressed in this article are solely those of the authors and do not necessarily represent those of their affiliated organizations, or those of the publisher, the editors and the reviewers. Any product that may be evaluated in this article, or claim that may be made by its manufacturer, is not guaranteed or endorsed by the publisher.

## Supplementary Material

Supplementary File 1 MPBCS Protocol.

Supplementary File 2 Extended Methods.

Supplementary Figure 1 Principal component analysis (PCA) from PAM50 gene expression data.

Supplementary Figure 2 Concordance of GSEA normalized enrichment scores (NES) in significantly enriched gene sets of MPBCS and TCGA*rf* stage II-III breast cancer tumors.

Supplementary Figure 3 Prognostic analysis of different TNBC subtypes (TNBC-6 and its derived TNBC-4 and Bareche-2018) in the MPBCS cohort.

Supplementary Table 1 GSEA and GSVA enriched terms for each subtype contrast in the MPBCS cohort.

Supplementary Table 2 Hazard ratios and statistical significance of Cox survival models for each prognostic classification tested.

Supplementary Table 3 MetaCore top ten pathways for each subtype contrast performed with all differentially expressed genes in common between TCGA and MPBCS, exclusively in MPBCS and exclusively in TCGA.
