# Supplementary material for: The Transcriptomic Portrait of Locally Advanced Breast Cancer and Its Prognostic Value in a Multi-Country Cohort of Latin American Patients
Source: Front Oncol. 2022 Mar 22;12:835626. doi: 10.3389/fonc.2022.835626 (PMC9007037; doi:10.3389/fonc.2022.835626)
Supplement: Supplementary File 1 — MPBCS Protocol. [file DataSheet_1.zip › Supplementary Figure 1.PDF]

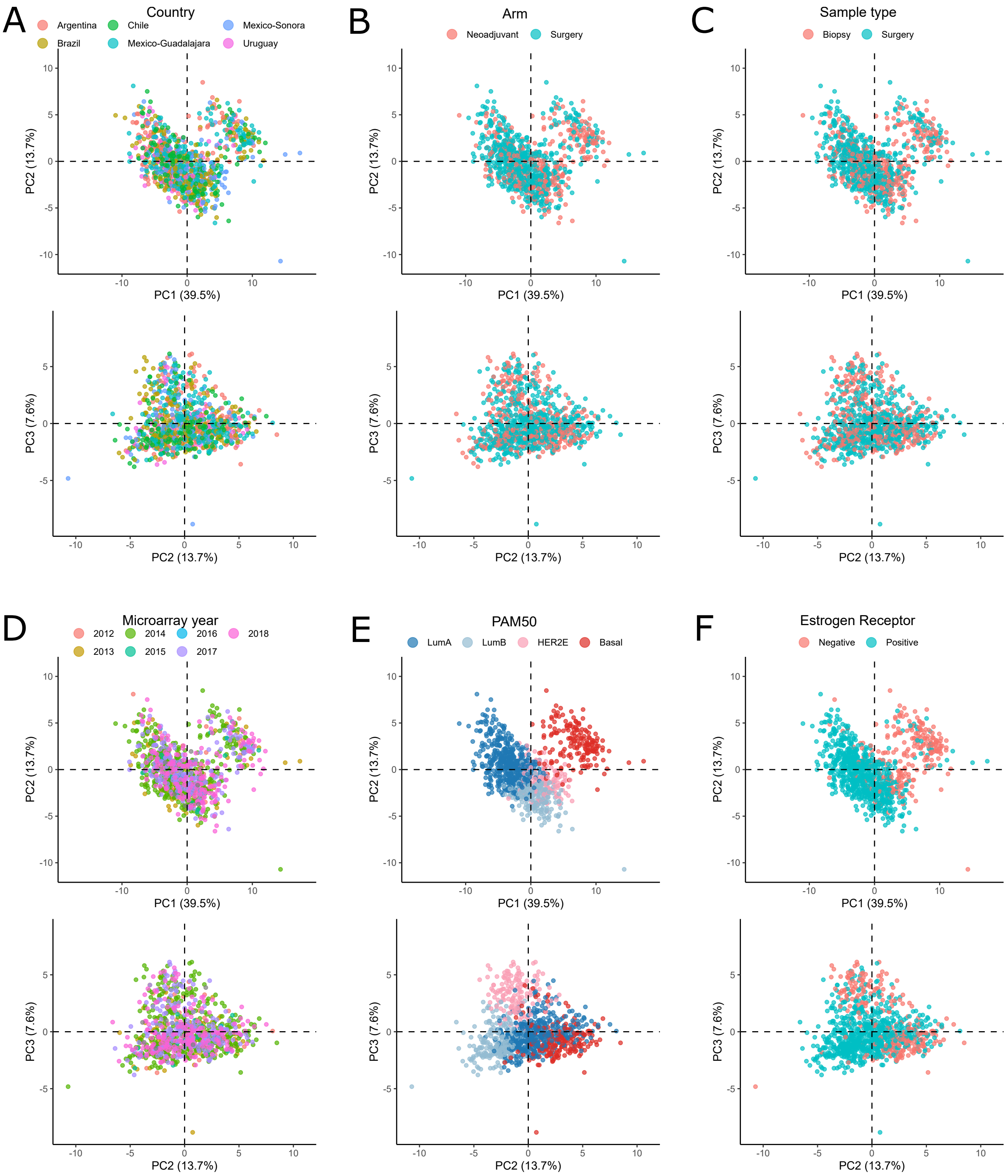

Additional Figure S1 - Principal component analysis of the expression of the PAM50 genes in the 1071 MPBCS patients.

PC1 vs PC2 scores (upper panel) and PC2 vs PC3 scores (lower panel) for A) country, B) arm of the study (primary surgery or neoadjuvancy), C) type of sample (biopsy or surgical specimen), D) year of microarray performance, E) PAM50 subtype, F) ER status.
