# Supplementary material for: The Transcriptomic Portrait of Locally Advanced Breast Cancer and Its Prognostic Value in a Multi-Country Cohort of Latin American Patients
Source: Front Oncol. 2022 Mar 22;12:835626. doi: 10.3389/fonc.2022.835626 (PMC9007037; doi:10.3389/fonc.2022.835626)
Supplement: Supplementary File 1 — MPBCS Protocol. [file DataSheet_1.zip › Supplementary Figure 2.PDF]

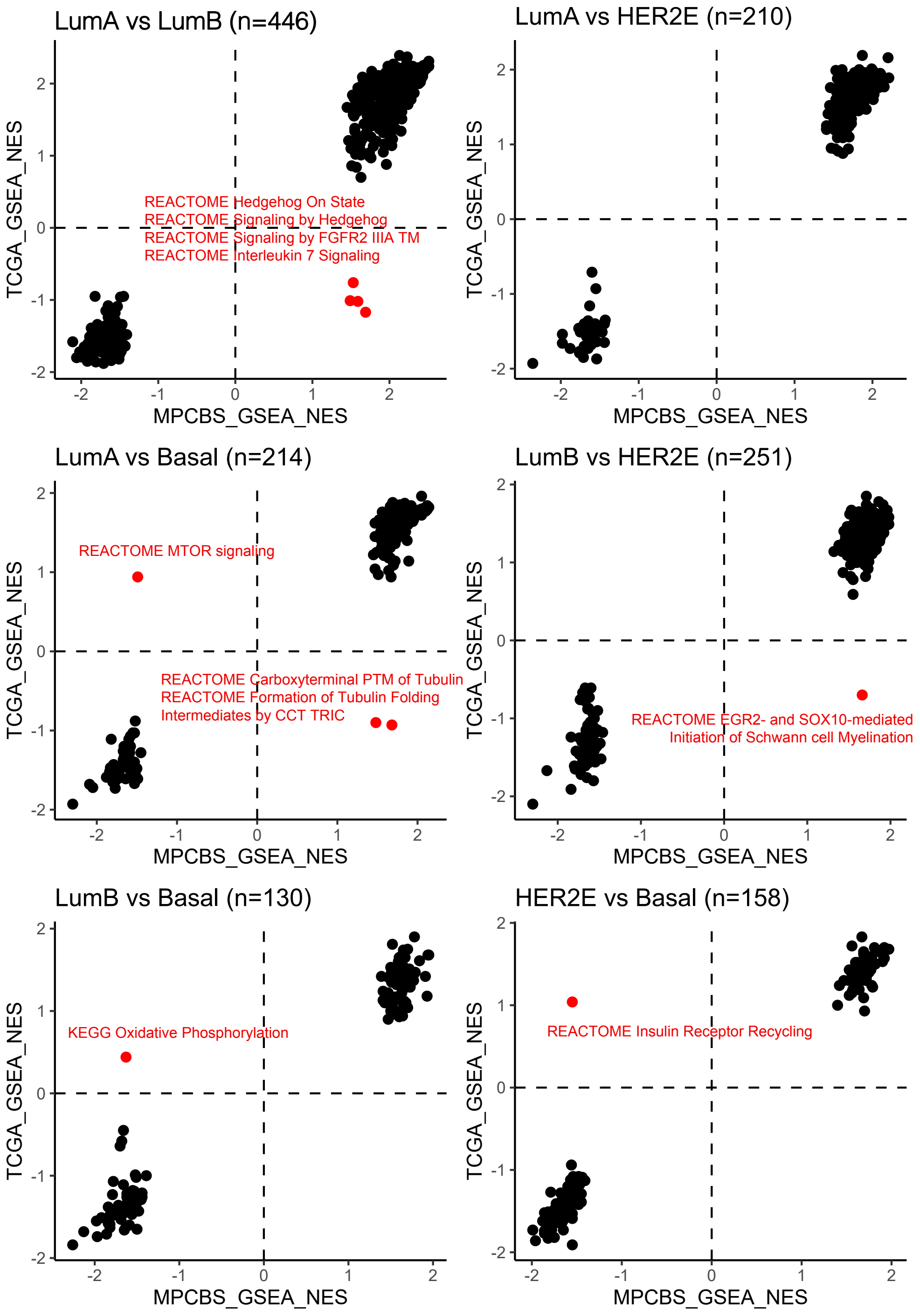

Supplementary Figure S2 - Concordance of GSEA normalized enrichment scores (NES) in significantly enriched gene sets of MPBCS and TCGArf stage II-III breast cancer tumors.
Each plot was done with the total significantly enriched of each contrast terms (n displayed) according to GSEA ($p<0{\cdot}05$) for the MPBCS cohort. Each dot corresponds to one enriched term. The names of the non-concordant terms (red dots) are mentioned next to their respective dots.
