# Supplementary material for: The Transcriptomic Portrait of Locally Advanced Breast Cancer and Its Prognostic Value in a Multi-Country Cohort of Latin American Patients
Source: Front Oncol. 2022 Mar 22;12:835626. doi: 10.3389/fonc.2022.835626 (PMC9007037; doi:10.3389/fonc.2022.835626)
Supplement: Supplementary File 1 — MPBCS Protocol. [file DataSheet_1.zip › Supplementary Figure 3.PDF]

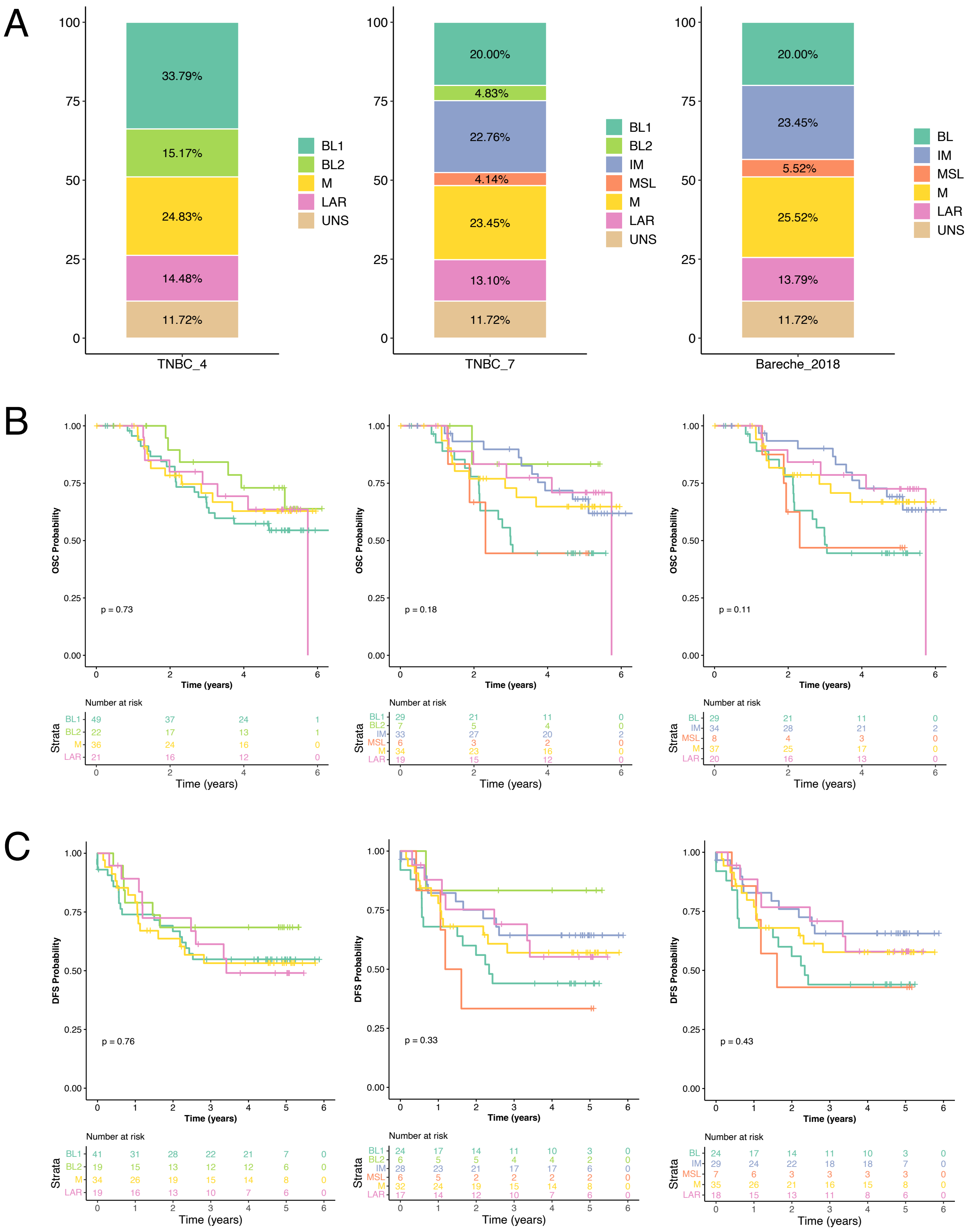

Supplementary Figure S3 – Prognostic analysis of different TNBC subtypes (TNBC-6 and its derived TNBC-4 and Bareche-2018) in the MPBCS cohort

A. Relative distribution of the different subtypes within each classification. BL, basal-like; M, mesenchymal; IM, immunomodulatory; MSL, mesenchymal stem-like; LAR, luminal androgen receptor. UNS represent tumors that could not be classified in either of the subtypes by Lehmann's algorithm.

B. Cancer-related OS (OSC) Kaplan Meier analysis for each of the classifications shown in A. Log-rank test p-value is shown in each graphic.

C. Disease-free survival (DFS) Kaplan Meier analysis for each of the classifications shown in A. Log-rank test p-value is shown in each graphic.
