# Supplementary material for: The Transcriptomic Portrait of Locally Advanced Breast Cancer and Its Prognostic Value in a Multi-Country Cohort of Latin American Patients
Source: Front Oncol. 2022 Mar 22;12:835626. doi: 10.3389/fonc.2022.835626 (PMC9007037; doi:10.3389/fonc.2022.835626)
Supplement: Supplementary File 2 — Extended Methods. [file DataSheet_2.pdf]

The transcriptomic portrait of locally advanced breast cancer and its prognostic value in a multi-country cohort of Latin American patients.

Supplementary File 2 - Extended Methods

Performance of the Ki67 % determination by immunochemistry

Given that there were no available proficiency assays regarding Ki67 detection at the moment of the analytical determinations, and that there was a lack of studies in Latin America that showed the local characteristics of Ki67 expression, we analyzed the performance of this determination among our institutions and countries. Due to the standardized pathological procedures applied in all the study, most preanalytical variables (e.g. buffered formalin fixation of 24+/-8 hs, cold ischemia time lower than 30 min, etc) were in accordance with the recent recommendations of the International Ki67 Working Group (IKWG, [1]). Having gene expression data available, we could demonstrate that Ki67% moderately correlated with *MKI67* gene expression levels (r=0.5) and we used this feature to determine if there was gross institutional or country bias. No remarkable institution- or country-effect was seen when each Ki67% was tagged with its corresponding *MKI67* expression levels (Figure 1). The median Ki67% of the entire cohort was of 30% (consistent with a bias towards advanced cases in this cohort) but by-country medians, as well as per-institution medians varied at a similar extent to what was previously described [2] (Figure 1).

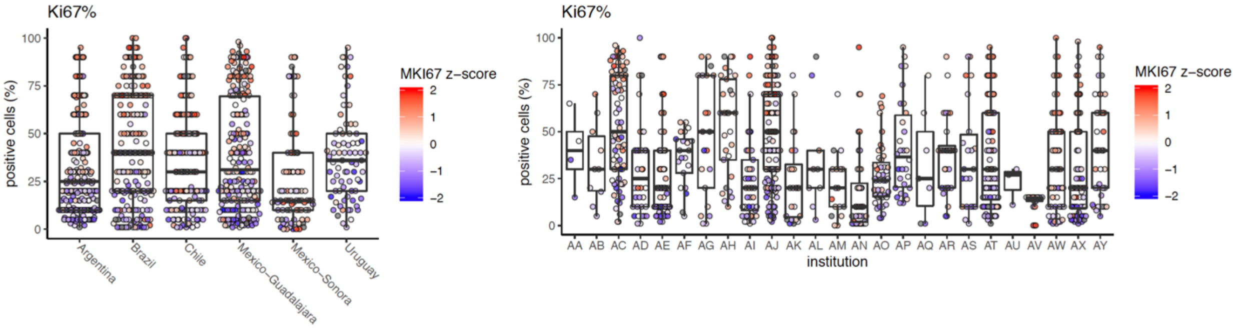

Figure 1 Distribution of KI67% among LACRN countries (left) and participating pathology laboratories (right). Each dot corresponds to one tumor, and the dot hue and shade indicates the corresponding z-score for *MKI67* gene expression in the corresponding tumor array. Red indicates overexpression and corresponds to higher Ki67%, blue represents underexpression and corresponds to lower Ki67%.

To define the best Ki67% threshold to distinguish LumB from LumA for descriptive purposes, we tested the performance of an overall cutoff value of 20% as well as the median of institutional Ki67% medians and the median of country medians. All thresholds had similar performances (i.e. similar AUC, not shown) to distinguish surrogate LumA and LumB tumors. Therefore, we decided to use a Ki67 of 20% as cutoff point for determining high or low proliferation, as recommended

by St Gallen's guidelines [3]. Interestingly, both MKI67 levels and Ki67% were also significantly correlated with non-LumA subtypes and were highest in Basal-like tumor (Figure 2). Figure 2 also shows that there is no remarkable bias in Ki67 % distribution among countries.

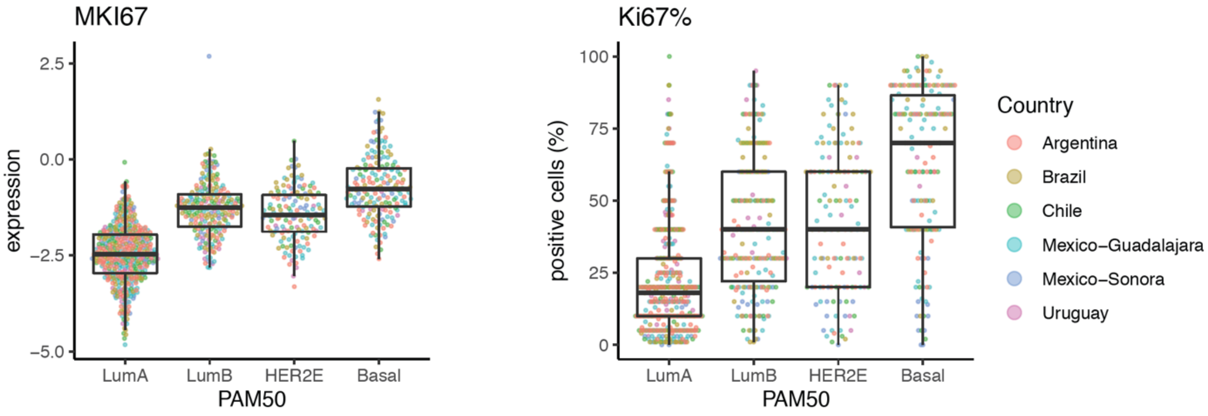

	LumA	LumB	HER2E	Basal
**LumA**		**−1.18**	**−1.02**	**−1.77**
**LumB**	**<0.001**		0.15	**−0.59**
**HER2E**	**<0.001**	0.11		**−0.74**
**Basal**	**<0.001**	**<0.001**	**<0.001**	

	LumA	LumB	HER2E	Basal
**LumA**		**0.41**	**0.37**	**0.16**
**LumB**	**<0.001**		0.89	**0.39**
**HER2E**	**<0.001**	0.952		**0.44**
**Basal**	**<0.001**	**<0.001**	**0.002**	

Figure 2. Distribution of *MKI67* gene expression (log ratio, left) and Ki67 protein (percentage of positive cells, right) according to the intrinsic subtype. Each dot represents one tumor, and dot shade depicts country of origin. No country bias was obvious in neither of the two markers. The table at the bottom of the *MKI67* plot shows for each pairwise comparison within the plot, the observed log-fold change of a differential expression analysis in the upper triangle and the adjusted p-values in the lower triangle. The table at the bottom of the Ki67% plot shows for each pairwise comparison the odds-ratio of a generalized linear model in the upper triangle and the adjusted p- values in the lower triangle.

Performance of microarray-based gene expression analysis

Two-color microarray analysis was performed in central molecular biology laboratories located in Argentina (Instituto Leloir), Brazil (Instituto de Câncer do Estado de Sao Paulo, Hospital do Câncer de Barretos, AC Camargo Cancer Center and Instituto Nacional de Câncer do Brasil), Mexico (University of Guadalajara and University of Sonora), Chile (Instituto de Salud Pública de Chile) and Uruguay (Instituto Pasteur). To verify the homogeneity of results from all centers, PCA and clustering analysis to check sources of expression variance across the samples was performed. Using only the PAM50 set of genes, no significant bias by country, arm of the study, type of sample (i.e. biopsy or surgical specimen) or year of microarray performance was seen on the first, second and third component (discussed in the manuscript text). In addition, unsupervised clustering of samples according to the expression levels of PAM50 genes shows a pattern similar to what was described in other cohorts, with basal and luminal samples conforming distinct groups, and HER2E samples clustering closer to the luminal samples (Figure 3).

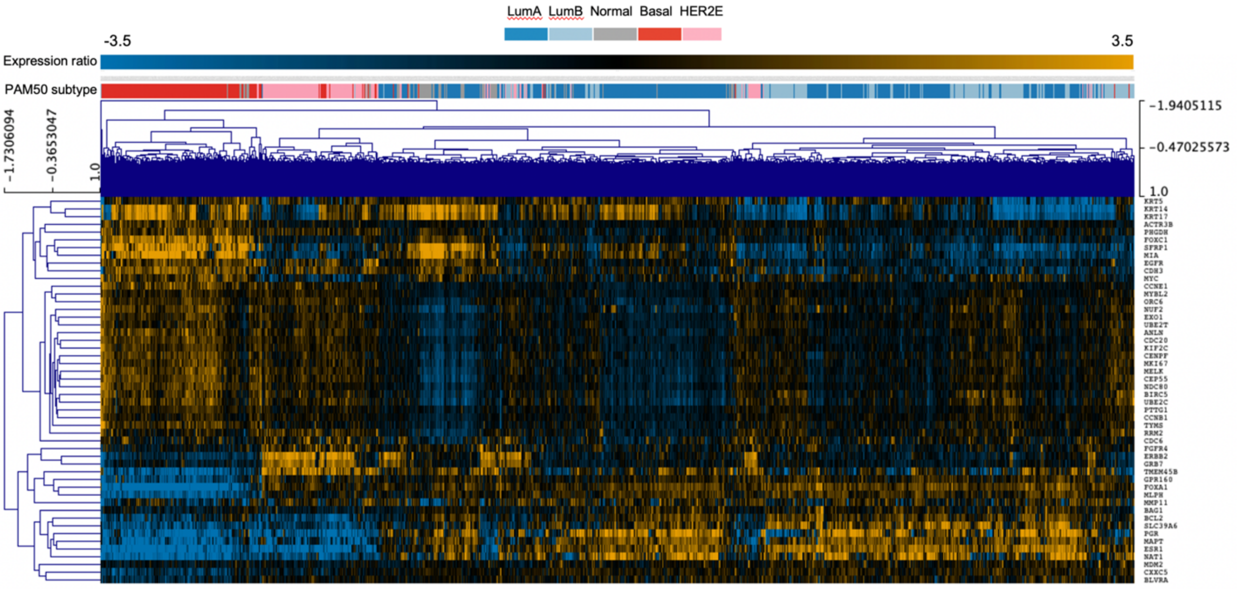

Figure 3 Heatmap of the unsupervised clustering of 1071 tumors (columns) according to PAM50 gene expression (rows). The category plot on the upper margin indicates the intrinsic subtype classification of each tumor.

PCA analysis conducted using the top 30% most variable genes (n=6257) also showed no significant bias by country, arm of the study, type of sample or year of microarray performance on the first and second component (Figure 4, panels A to D), and discriminated PAM50 subtypes and ER status (Figure 4E and F). However, the analysis of the third PCA component (5.5% of total variation) revealed a bias by country, also perceived in the year in which microarrays were run (Figure 4A and D, right panels), which also varied by country. This finding prompted us to perform a batch correction, i.e. prior to averaging probe expression by gene symbol, two batches based on processing dates (i.e. before or after September 2017) were defined for the whole cohort. Probe expression data was then normalized to equalize median-absolute values within each cohort and adjusted for batch effects using ComBat.

References

1. Nielsen TO, Leung SCY, Rimm DL, Dodson A, Acs B, Badve S, et al. Assessment of Ki67 in Breast Cancer: Updated Recommendations From the International Ki67 in Breast Cancer Working Group. J Natl Cancer Inst. 2021;113:808–19.
2. Polley MYC, Leung SCY, Gao D, Mastropasqua MG, Zabaglo LA, Bartlett JMS, et al. An international study to increase concordance in Ki67 scoring. Mod Pathol. 2015;28:778–86.
3. Goldhirsch A, Winer EP, Coates AS, Gelber RD, Piccart-Gebhart M, Thürlimann B, et al. Personalizing the treatment of women with early breast cancer: highlights of the St Gallen International Expert Consensus on the Primary Therapy of Early Breast Cancer 2013. Ann Oncol. 2013;24:2206–23.

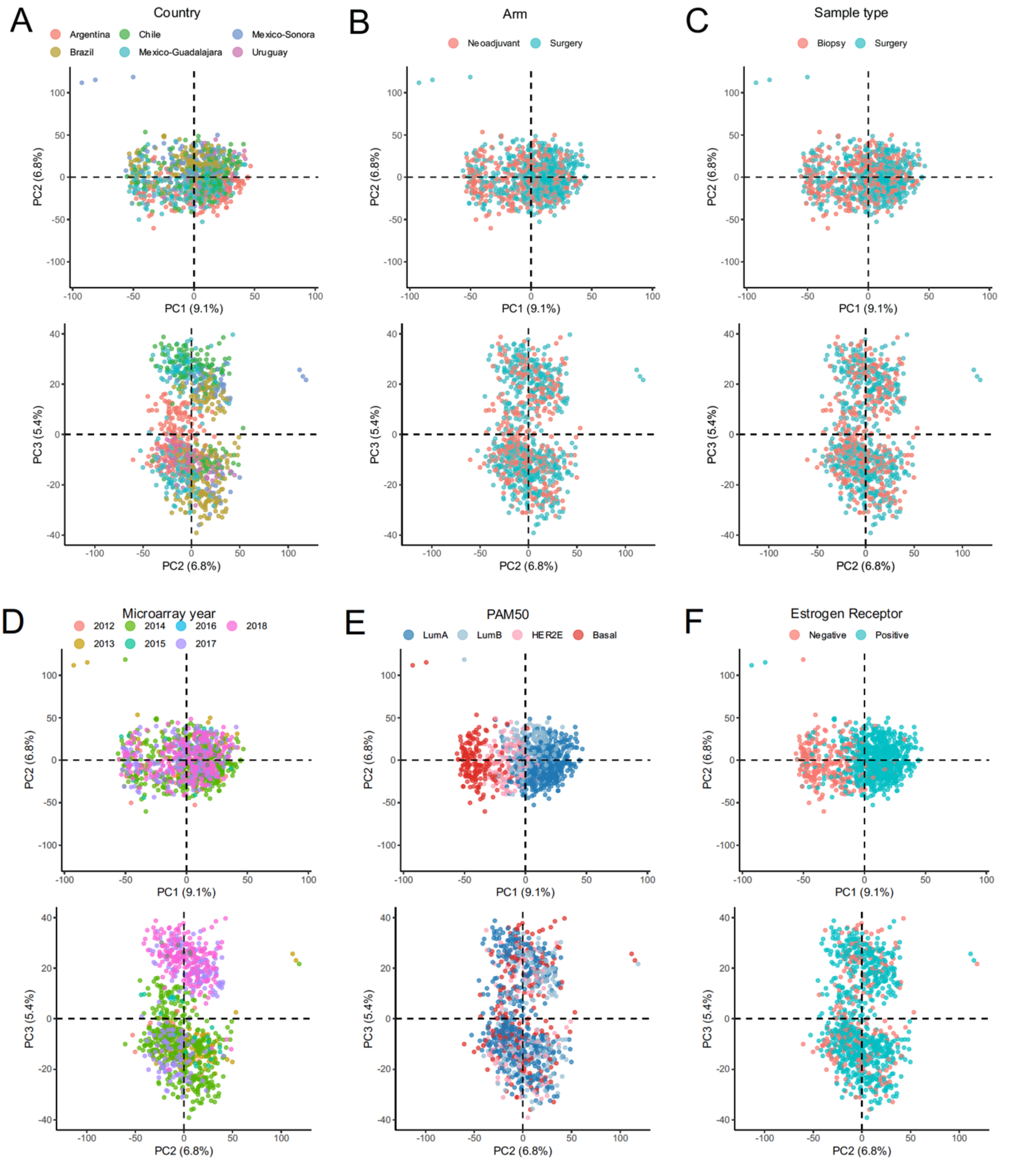

Figure 4. Principal component analysis of the expression of the 30% genes with highest variance in the 1071 MPBCS tumors

PC1 vs PC2 scores (upper panel) and PC2 vs PC3 scores (lower panel) for A) country, B) arm of the study (primary surgery or neoadjuvacy), C) type of sample (biopsy or surgical specimen), D) year of microarray performance, E) PAM50 subtype, F) ER status. Each dot represents one tumor
